# Fatal progression of experimental visceral leishmaniasis is associated with intestinal parasitism and secondary infection by commensal bacteria, and is delayed by antibiotic prophylaxis

**DOI:** 10.1371/journal.ppat.1008456

**Published:** 2020-04-13

**Authors:** Michael D. Lewis, Andrea Paun, Audrey Romano, Harry Langston, Charlotte A. Langner, Ian N. Moore, Kevin W. Bock, Amanda Fortes Francisco, Jason M. Brenchley, David L. Sacks

**Affiliations:** 1 Department of Infection Biology, London School of Hygiene and Tropical Medicine, Keppel Street, London, United Kingdom; 2 Laboratory of Parasitic Diseases, National Institute of Allergy and Infectious Diseases, Bethesda, Maryland, United States of America; 3 Barrier Immunity Section, Laboratory of Viral Diseases, National Institute of Allergy and Infectious Diseases, Bethesda, Maryland, United States of America; 4 Infectious Disease Pathogenesis Section, Comparative Medicine Branch, National Institute of Allergy and Infectious Diseases, Bethesda, Maryland, United States of America; Uniformed Services University of the Health Sciences, UNITED STATES

## Abstract

*Leishmania donovani* causes visceral leishmaniasis (VL), which is typically fatal without treatment. There is substantial variation between individuals in rates of disease progression, response to treatment and incidence of post-treatment sequelae, specifically post-kala-azar dermal leishmaniasis (PKDL). Nevertheless, the majority of infected people are asymptomatic carriers. Hamsters and mice are commonly used as models of fatal and non-fatal VL, respectively. Host and parasite genetics are likely to be important factors, but in general the reasons for heterogeneous disease presentation in humans and animal models are poorly understood. Host microbiota has become established as a factor in cutaneous forms of leishmaniasis but this has not been studied in VL. We induced intestinal dysbiosis in mice and hamsters by long-term treatment with broad-spectrum antibiotics in their drinking water. There were no significant differences in disease presentation in dysbiotic mice. In contrast, dysbiotic hamsters infected with *L*. *donovani* had delayed onset and progression of weight loss. Half of control hamsters had a rapid progression phenotype compared with none of the ABX-treated animals and the nine-month survival rate was significantly improved compared to untreated controls (40% vs. 10%). Antibiotic-treated hamsters also had significantly less severe hepatosplenomegaly, which was accompanied by a distinct cytokine gene expression profile. The protective effect was not explained by differences in parasite loads or haematological profiles. We further found evidence that the gut-liver axis is a key aspect of fatal VL progression in hamsters, including intestinal parasitism, bacterial translocation to the liver, malakoplakia and iron sequestration, none of which occurred in non-progressing murine VL. Diverse bacterial genera were cultured from VL affected livers, of which *Rodentibacter* was specifically absent from ABX-treated hamsters, indicating this pathobiont may play a role in promoting disease progression. The results provide experimental support for antibiotic prophylaxis against secondary bacterial infections as an adjunct therapy in human VL patients.

## Author summary

Visceral leishmaniasis (VL) is a potentially fatal neglected infectious disease that is widespread in South Asia, East Africa, South America and the Mediterranean region. VL is caused by infections with the protozoan parasite *Leishmania donovani*. Infected people may remain asymptomatic or progress, at variable rates, to develop VL disease affecting the liver, spleen and blood. The reasons why clinical outcomes vary so much is not well understood. We decided to investigate whether a host’s gut microbiota might be a relevant factor. We treated mice and hamsters with antibiotics to disrupt their gut microbiota and then infected them with *L*. *donovani*. No effect was observed in mice, however, treated hamsters had slower onset and progression of VL, less severe enlargement of the liver and spleen, and had a reduced mortality rate. This was accompanied by alterations in the levels of key immune response factors. We also found novel aspects of symptomatic VL in hamsters relating to the gut and its microbiota. Specifically, the gut tissues themselves were parasitized by *L*. *donovani* and liver tissues became co-infected with both *L*. *donovani* and Gram negative bacteria originating from the gut. Overall, our findings support the inclusion of anti-bacterial therapy as part of VL treatment strategies.

## Introduction

Kala-azar or visceral leishmaniasis (VL) is caused by infection with members of the *Leishmania donovani* species complex (*L*. *donovani*, *L*. *infantum/chagasi*). These parasitic protozoa are transmitted to humans and reservoir mammals during blood feeding by infected female sand flies. VL is endemic in large parts of South Asia, East Africa, Latin America and the Mediterranean region. A recent report by WHO on the 14 high-burden countries (>100 cases/year) shows a decrease in overall cases reported, down from an average of 58,000 new cases reported per year between 2004–2008, to 30,758 new cases in 2014, with underreporting estimated to be from 1.2- to 4-fold [1]. Symptomatic VL is typically characterised by gross enlargement of the spleen and liver, pancytopaenia and hypergammaglobulinaemia, and is considered fatal without anti-parasitic chemotherapy [2]. This pathology is associated with high parasite loads and chronic inflammatory responses in the visceral organs, lymph nodes and bone marrow. Death is usually attributed to factors secondary to *L*. *donovani* infection, including immunosuppression, haemorrhages and opportunistic infections [3]. The rate of progression varies substantially between individuals, as does the incidence of post-kala-azar dermal leishmaniasis (PKDL, a post-treatment complication) in different regions [4]. Moreover, 80–95% of *L*. *donovani* infections are thought to be subclinical or asymptomatic [5, 6]. This heterogeneity of VL outcomes is thought to reflect both genetic and environmental factors [7–9]. Some specific determinants of susceptibility have been identified, for example host nutritional status [10] and polymorphisms in the *HLA* locus [11], however, the mechanistic basis of fatal progression is poorly understood.

Hamsters and susceptible mouse strains (e.g. C57BL/6, BALB/c) are the most widely used experimental models of *L*. *donovani* infection. The hamster model recapitulates many features of symptomatic human VL, including hepatosplenomegaly and haematological defects. It is characterised by progressively increasing parasite loads in target organs and develops into a cachexia-like syndrome, which becomes fatal after several months of infection [12, 13]. Whether the causes of death in hamsters are similar to those in human VL cases is unclear. Of note, opportunistic pathogens equivalent to those affecting human patients are absent from vivaria with specific pathogen-free (SPF) environments. Murine VL is not fatal; it features stable, chronic infection of the spleen causing splenomegaly but parasitism of the liver is slowly resolved by effective immune responses [14, 15]. One reason for the effective control of *L*. *donovani* infection in mice compared to hamsters is the relatively enhanced induction of protective iNOS activity in infected murine macrophages [16]. Hamster macrophages appear to be hypo-responsive to IFN-γ and are more likely to adopt a permissive state characterised by high STAT6-dependent arginase I expression [17]. However, it is not clear whether the inter-species difference in this pathway can fully explain the lethality of *L*. *donovani* infection in hamsters.

This study aimed to address the hypothesis that the host microbiota is a factor affecting disease outcomes in experimental VL models. Commensal skin bacteria have been shown to influence the host-parasite interaction and pathology in models of cutaneous leishmaniasis (CL). Specifically, germ-free mice inoculated with *L*. *major* or *L*. *amazonensis* have been reported to develop smaller skin lesions [18–20], although others have observed larger lesions [21]. These data generally support a pro-inflammatory function for the microbiota in CL. To our knowledge there are no comparable data for VL. A key difference is that unlike in CL, the pathologies associated with chronic *L*. *donovani* infection do not occur in sites where commensals are normally present. Nevertheless, the impact of the gut microbiota on immune responses in distant sites is becoming increasingly apparent [22].

We disrupted the normal intestinal microbiota of mice and hamsters using antibiotics and studied the effect on *L*. *donovani* infection. Murine VL was unaffected, however, a significant improvement of outcomes was observed in hamsters. Fatal progression was accompanied by evidence of bacterial translocation from the gut to the liver, which exacerbated hepatic pathology. The results provide experimental support for clinical trials incorporating antibiotic prophylaxis against secondary bacterial infections as an adjunct therapy in human VL patients.

## Results

### Effect of antibiotic-induced dysbiosis on VL in mice

**Fig 1:**
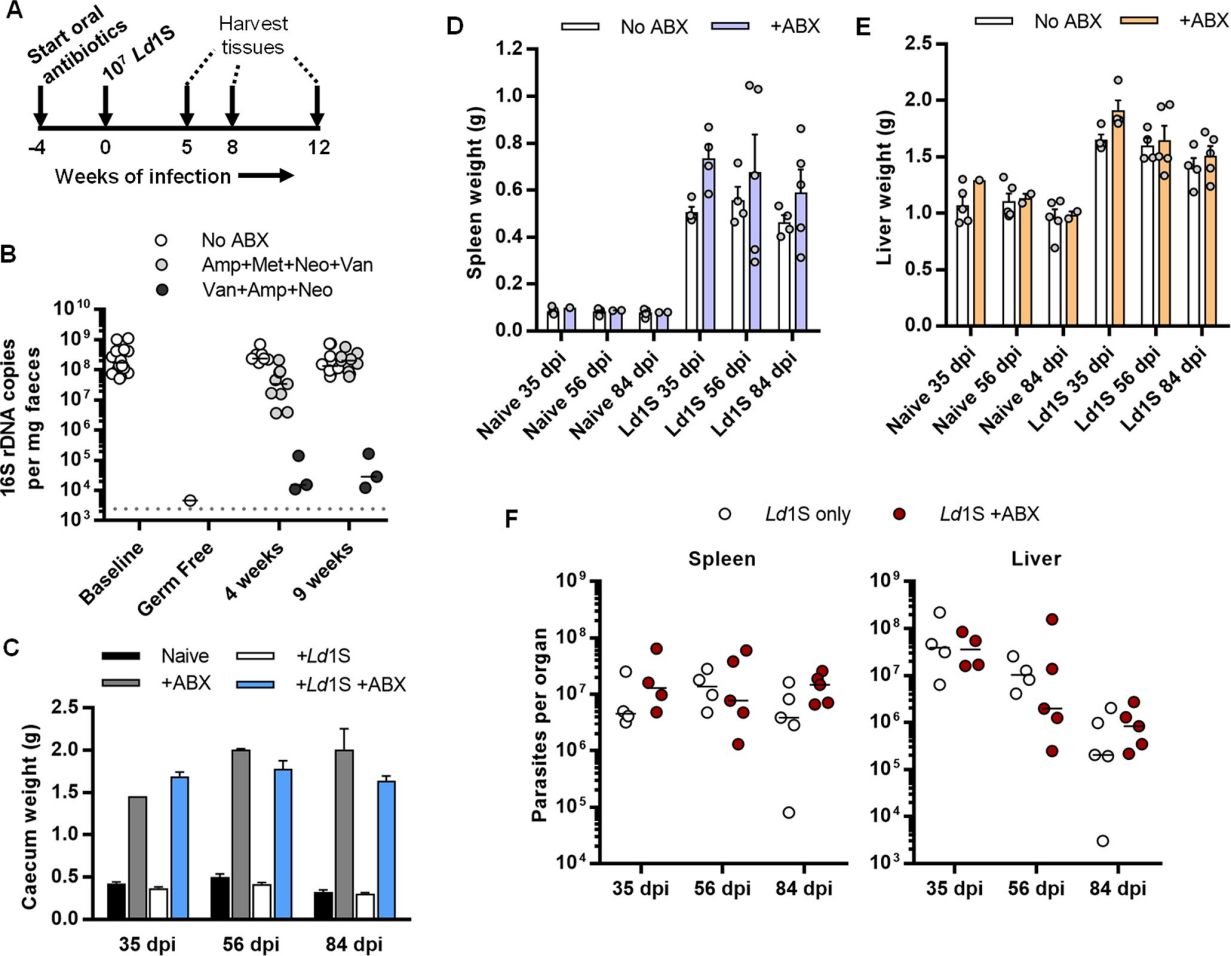
Effects of oral antibiotic treatment on outcomes in a mouse model of non-progressing visceral leishmaniasis. (A) Experimental design; (B) qPCR analysis of bacterial 16S rDNA content in mouse faecal pellets at different times before (n = 21) and during oral antibiotic regimens (n = 3–9), including a germ-free control (n = 1). Lines show medians and dashed line shows the detection limit (mean + 2 standard deviations of negative extraction controls); (C, D, E, F) Caecum (C), spleen (D) and liver (E) weights, and parasite loads (F) measured at necropsy at the indicated times after infection with *Leishmania donovani* strain 1S (*Ld*1S) and maintained on drinking water containing antibiotic cocktail (ABX) compared with naïve controls and no ABX controls, dpi, days post-infection, n = 2–5 per group.

To investigate a potential role for the host microbiota in the mouse model of VL, we treated C57BL/6 mice with broad-spectrum antibiotics via their drinking water prior to i.v. injection with 10^7^ *L*. *donovani* strain 1S (*Ld*1S) metacyclic promastigotes and throughout the following infection (Fig 1A). We observed that a cocktail of ampicillin, vancomycin and neomycin (ABX) led to a strong and sustained depletion of gut microbiota, as measured by the 16S rDNA copy number in faeces (Fig 1B). We tested the effect of metronidazole as a fourth antibiotic [23] but this led to reduced fluid intake and was associated with less efficient microbiota depletion. Profound dysbiosis in ABX-treated animals was evidenced macroscopically by gross enlargement of the caecum, a result of the loss of bacterial species required for normal fermentation of dietary carbohydrates (Fig 1C). This effect was equivalent in both control uninfected and *Ld*1S-infected animals.

ABX-treated mice had a larger average spleen size at 35, 56 and 84 days after *Ld*1S infections but this difference was not significant due to substantially increased variation in the treated groups (Fig 1D). The increased splenomegaly observed in a subset of individual ABX-treated mice may indicate an effect associated with differential dysbiosis. No effect was observed with respect to hepatomegaly (Fig 1E) and ABX-treatment did not lead to differences in spleen or liver weights in uninfected controls (Fig 1D and 1E). Parasite loads in the liver and spleen were equivalent in both groups and conformed to the expected pattern of stable chronic infection in the spleen and gradually declining parasite loads in the liver (Fig 1F). Overall, these data show that disruption of the normal intestinal microbiota had no significant impact on the mouse model of non-progressing VL.

### Antibiotic-induced dysbiosis ameliorates VL in hamsters

**Fig 2:**
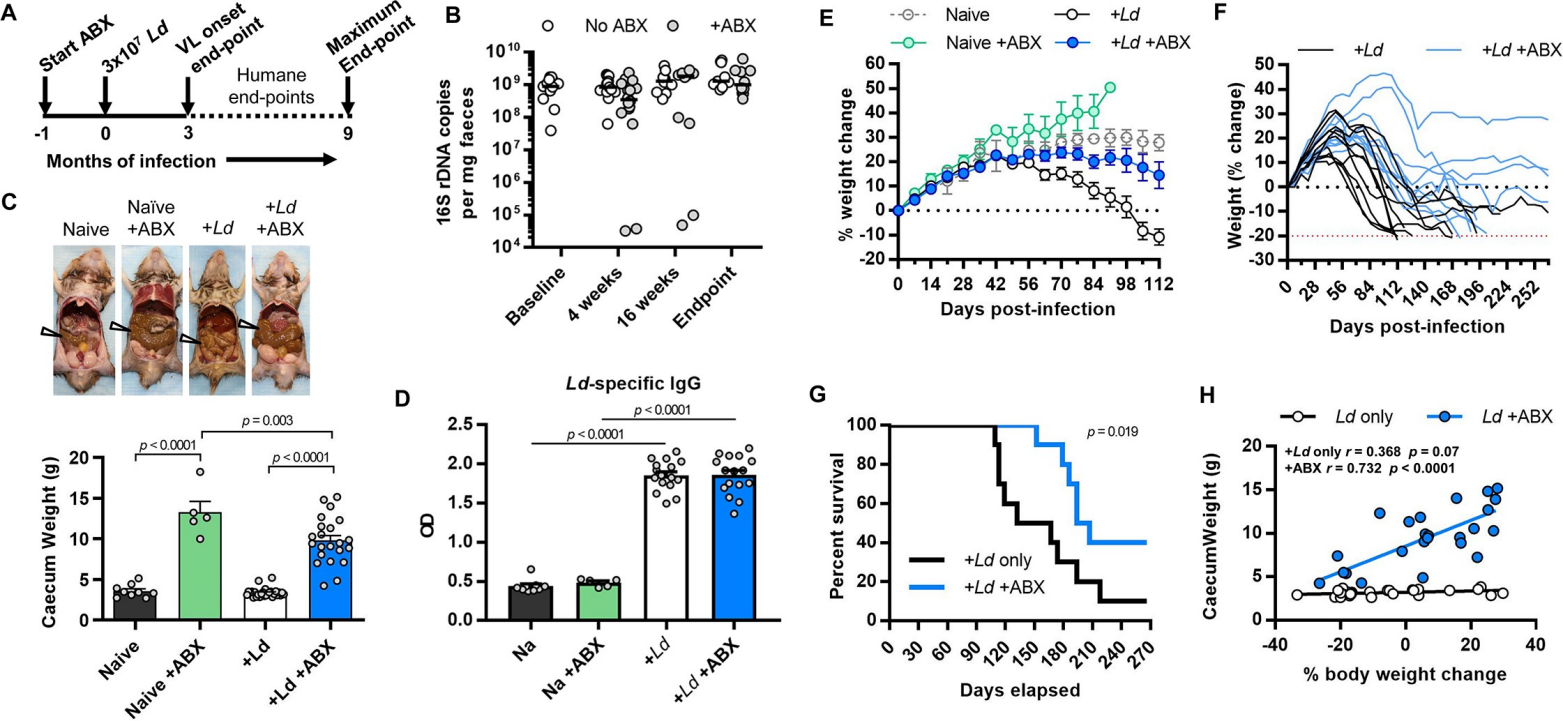
Oral antibiotic treated hamsters have delayed VL disease progression and reduced mortality. (A) Experimental design; (B) qPCR analysis of bacterial 16S rDNA content in hamster faecal pellets at different times (x-axis) before and during oral antibiotic treatment (ABX); *Leishmania donovani* (*Ld*) infections were initiated at 4 weeks of ABX treatment. Lines show medians. (C) Representative images of viscera at humane end-point and caecum weights at 12 weeks post-infection in *Leishmania donovani* (*Ld*)-infected hamsters maintained on drinking water containing antibiotic cocktail (ABX) compared with naïve controls and no ABX controls; arrows indicate caecum. (D) *L*. *donovani* whole cell lysate-reactive IgG antibody titres measure by ELISA (OD, optical density), compared by one-way ANOVA. (E,F) Weight change over time, data shown for means (E) and individual animals (F) where dashed red line marks humane end-point. (G) Survival curve for *Ld*-infected hamsters treated with ABX or not, Gehan-Breslow-Wilcoxon test *p* = 0.019. (H) Pairwise comparison and Pearson correlation tests of individual animal’s caecum weight and post-infection change in body weight. Experiments using VL onset end point (B, C, D, E, H) n = 5–16 per group; experiments using humane/maximum end-points (F, G), n = 10 per group.

Unlike the mouse model, our standard model of hamster VL (3 x 10^7^ *L*. *donovani* 1S metacyclic promasitgotes injected intra-cardially) is progressive and usually results in death within 6 months. This model is considered to better reflect symptomatic human VL than the mouse [24]. We therefore conducted experiments to compare VL outcomes in ABX-treated and untreated control *Ld*1S-infected hamsters up to a maximum of nine months (Fig 2A). Continuous ABX treatment led to a more moderate faecal microbiota depletion than was seen in mice and the 16S rDNA copy numbers returned to normal levels by the time that the hamster’s humane end-points were reached (Fig 2B). Nevertheless, massive enlargement of the caeca of ABX-treated hamsters indicated that dysbiosis was maintained during the experiments (Fig 2C).

Hamsters in both experimental groups were susceptible to infection and had similar *L*. *donovani*-specific IgG titres (Fig 2D). In the hamster VL model, weight loss is considered a proxy for disease progression, and we designated a 20% loss in original body weight as the humane endpoint. As expected, the onset of VL-related weight loss in hamsters infected with *Ld*1S and given control water occurred at 2 months post-infection (p.i.) (Fig 2E). The progression of disease occurred very rapidly in half of these animals, with terminal humane endpoints occurring after 112–132 days of infection (Fig 2F and 2G). End-points were reached by the remaining slower-progressing animals between 6 and 8 months p.i. (Fig 2F). This variability is likely attributable to the outbred nature of experimental hamsters. The onset of weight loss was significantly delayed in ABX-treated hamsters (Fig 2E) and there was a strong negative correlation between caecum enlargement (dysbiosis) and the severity of weight loss (disease progression) specifically in this group and not untreated controls (Fig 2H). Weight loss was less marked in male hamsters but ABX treatment still had a significant effect on weight profiles (S1 Fig). The rate of survival for nine months was significantly better (40% vs. 10%, *p* = 0.036, log-rank test) (Fig 2G) and those treated hamsters that did succumb to the infection were all slow-progressors (Fig 2F). Therefore, ABX-treatment was associated with a dramatic improvement of outcomes in *Ld*1S-infected hamsters.

### Infection and tissue remodelling

**Fig 3:**
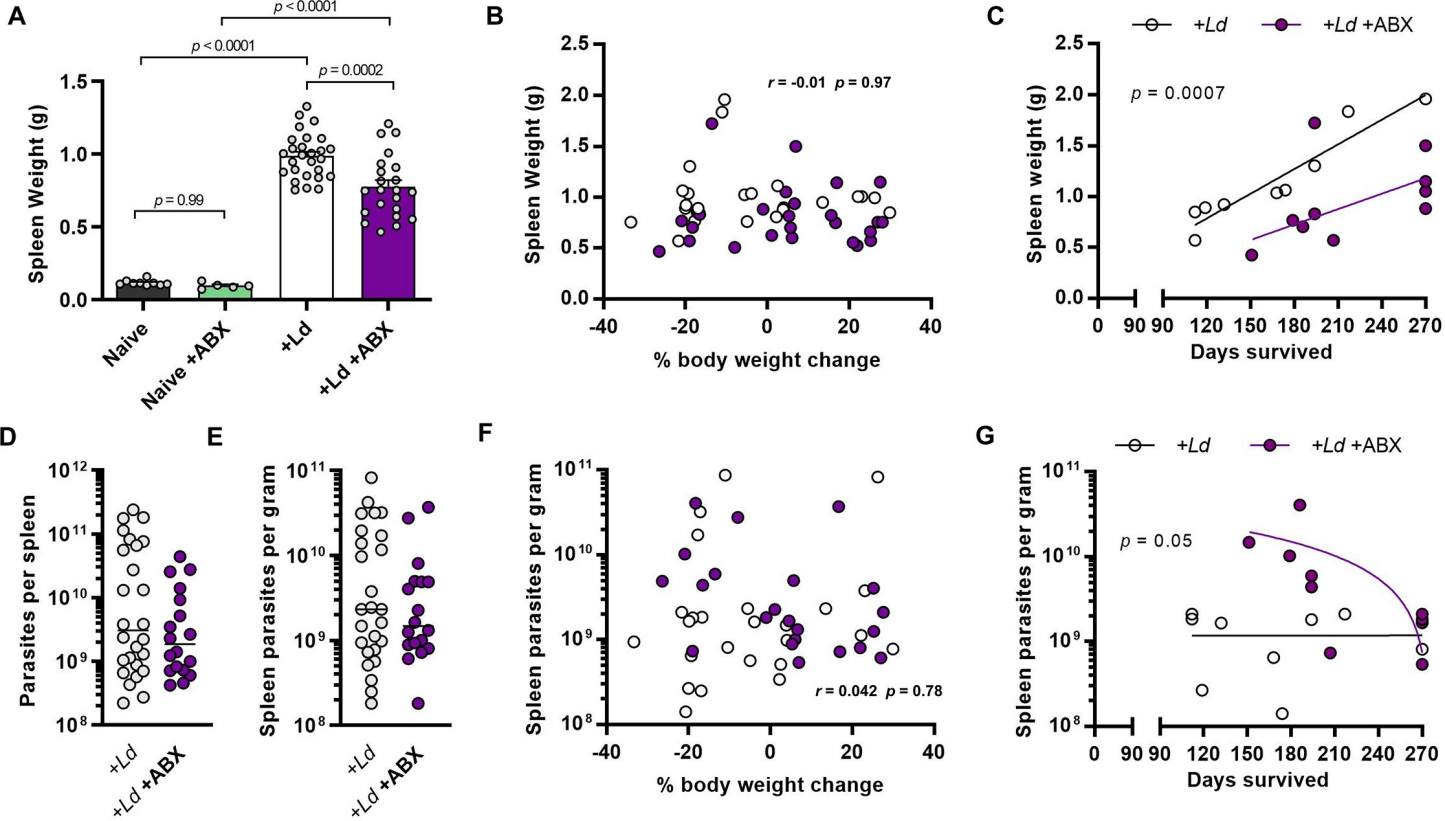
Prolonged survival in antibiotic treated hamsters is associated with less severe splenomegaly despite equivalent or higher parasite loads. (A) Spleen weights of hamsters at 12 weeks post-infection (p.i.) with *L*. *donovani* and given drinking water containing antibiotic cocktail (ABX) compared with uninfected naïve and untreated controls; *p*-values are from one way ANOVA. (B) Pairwise comparison and Pearson correlation test of splenomegaly severity and change in body weight (proxy for disease progression) at 12 weeks p.i. (C) Pairwise comparison between splenomegaly severity and time to humane end-point (fatal progression); *p*-value is from comparison of linear regression slope elevations. (D,E) Total parasite loads (D) and infection intensities (E) in spleens after 12 weeks of infection, measured by limiting dilution ex vivo culture assay. (F) Pairwise comparison and Pearson correlation test of spleen parasite densities and change in body weight (proxy for disease progression) at 12 weeks p.i. (G) Pairwise comparison between spleen parasite density and time to humane end-point (fatal progression); *p*-value is from comparison of linear regression slope elevations. In B-G data points are for individual hamsters treated with oral antibiotics (filled circles) and untreated controls (open circles). Analysis using VL onset end point (A,D,E), n = 5–27 per group; analysis using humane/maximum end-points (C, G), n = 9–10 per group; analysis using all endpoints (B,F), n = 22–25 per group.

Enlargement of the spleen is a key feature of human VL and splenic aspirates are widely used to diagnose VL patients [25]. At 12 weeks p.i. (a stage when control infected hamsters have become symptomatic), we found that ABX-treated hamsters had significantly less severe splenomegaly than hamsters given control water (Fig 3A), which was in keeping with the weight loss and mortality results. Splenomegaly did not correlate with overall weight loss at this stage of infection (Fig 3B). Confining the analysis to hamsters that were allowed to reach their humane endpoint, we found that the spleens of longer surviving animals were typically larger than in fast progressing ones and were significantly smaller in the ABX-treated group (Fig 3C). Splenic remodelling is therefore not likely to be a direct cause of fatal disease progression in this model. We reasoned that improved outcomes in ABX-treated hamsters might be associated with lower burden of *L*. *donovani* infection, but surprisingly this was not the case. Spleen parasite loads at 12 weeks p.i. were not significantly different from control animals either in terms of total number of parasites (Fig 3D) or the density of infection (Fig 3E). Parasite loads did not show any association with weight loss at this time point (Fig 3F, S2 Fig) or with survival time in the longer term (Fig 3G, S2 Fig), suggesting that other factors drive disease progression.

**Fig 4:**
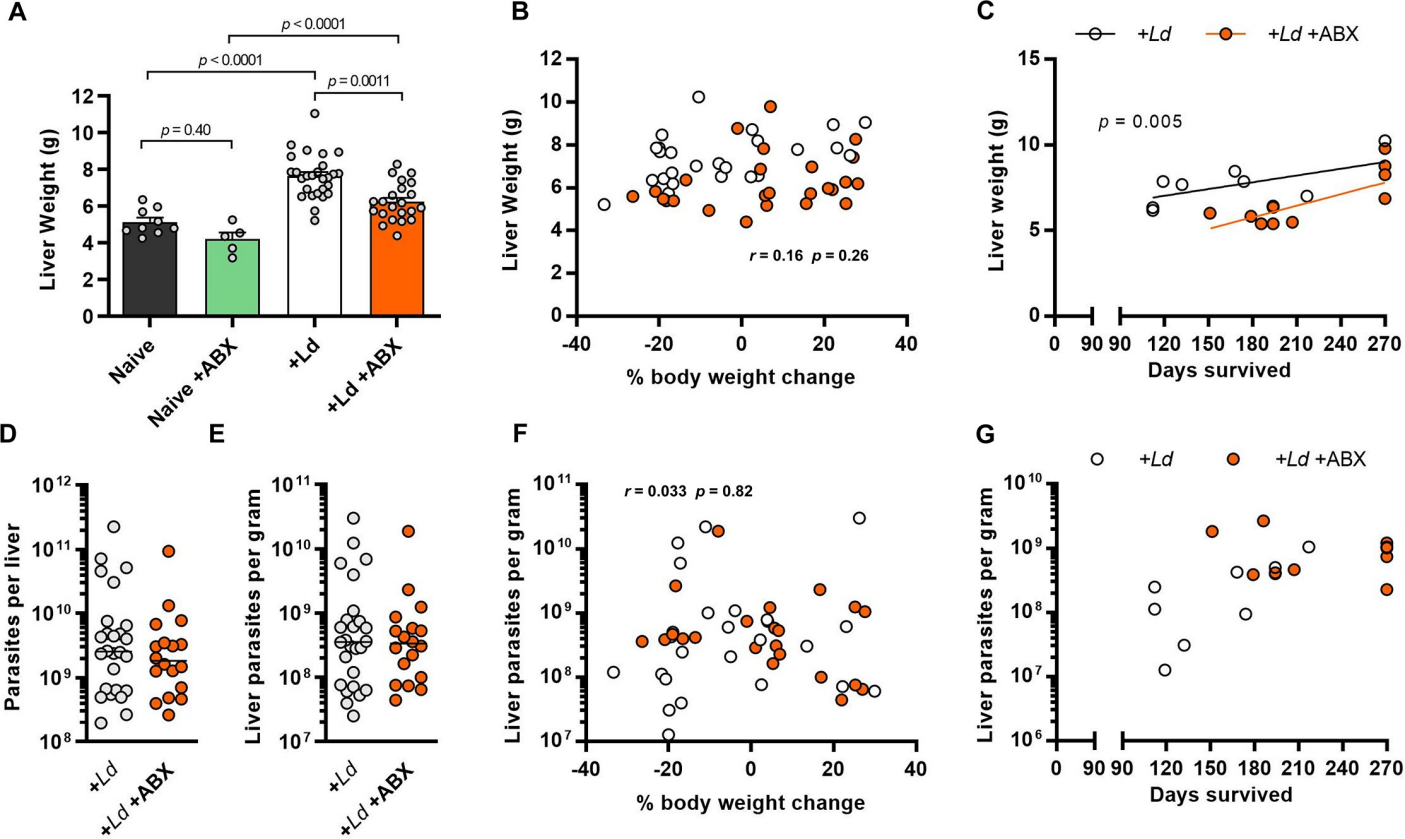
Prolonged survival in antibiotic treated hamsters is associated with less severe hepatomegaly despite equivalent parasite loads. (A) Liver weights of hamsters at 12 weeks post-infection (p.i.) with *L*. *donovani* and given drinking water containing antibiotic cocktail (ABX) compared with uninfected naïve and untreated controls; *p*-values are from one way ANOVA. (B) Pairwise comparison and Pearson correlation test of hepatomegaly severity and change in body weight (proxy for disease progression) at 12 weeks p.i. (C) Pairwise comparison between hepatomegaly severity and time to humane end-point (fatal progression); *p*-value is from comparison of linear regression slope elevations. (D,E) Total parasite loads (D) and infection intensities (E) in livers after 12 weeks of infection, measured by limiting dilution ex vivo culture assay. (F) Pairwise comparison and Pearson correlation test of liver parasite densities and change in body weight (proxy for disease progression) at 12 weeks p.i. (G) Pairwise comparison between liver parasite density and time to humane end-point (fatal progression). In B-G data points are for individual hamsters treated with oral antibiotics (filled circles) and untreated controls (open circles). Analysis using VL onset end point (A,D,E), n = 5–27 per group; analysis using humane/maximum end-points (C, G), n = 9–10 per group; analysis using all endpoints (B,F), n = 22–25 per group.

Failure to control *L*. *donovani* levels in the liver and enlargement of this organ are also key features of both human VL and the fatal hamster model, whereas liver infection and pathology resolve slowly in the non-progressing mouse model [12–14]. We found that hepatomegaly at 12 weeks p.i. was significantly less severe in ABX-treated hamsters than in untreated controls (Fig 4A). There was no association between liver weight and overall weight loss, i.e. disease progression, at this time point (Fig 4B). In the longer term, confining the analysis to hamsters that had reached their humane endpoint, animals that suffered rapid fatal progression tended to have less severe hepatomegaly in both experimental groups (Fig 4C). Irrespective of survival time, ABX-treated animals had significantly smaller livers than untreated controls (Fig 4C). In terms of parasite loads the findings mirrored the results for the spleen. Total numbers of liver parasites (Fig 4D) and parasite density (Fig 4E) were equivalent at 12 weeks p.i. in the ABX-treated and control groups, and these were not associated with disease progression rate (Fig 4F, S2 Fig). Liver infection was less intense in more rapidly progressing hamsters (Spearman rank correlation *r* = 0.59, *p* = 0.028), but overall this parameter was not significantly different between treated and untreated groups at survival end points (Fig 4G).

Overall, these data demonstrate that slower progression of VL and reduced tissue remodelling in ABX-treated hamsters is not associated with lower parasite burdens in the spleen or liver. More generally, they also indicate that neither parasite loads in, nor enlargement of, these organs are significant correlates of fatal VL progression.

### Disruption of haematological homeostasis

**Fig 5:**
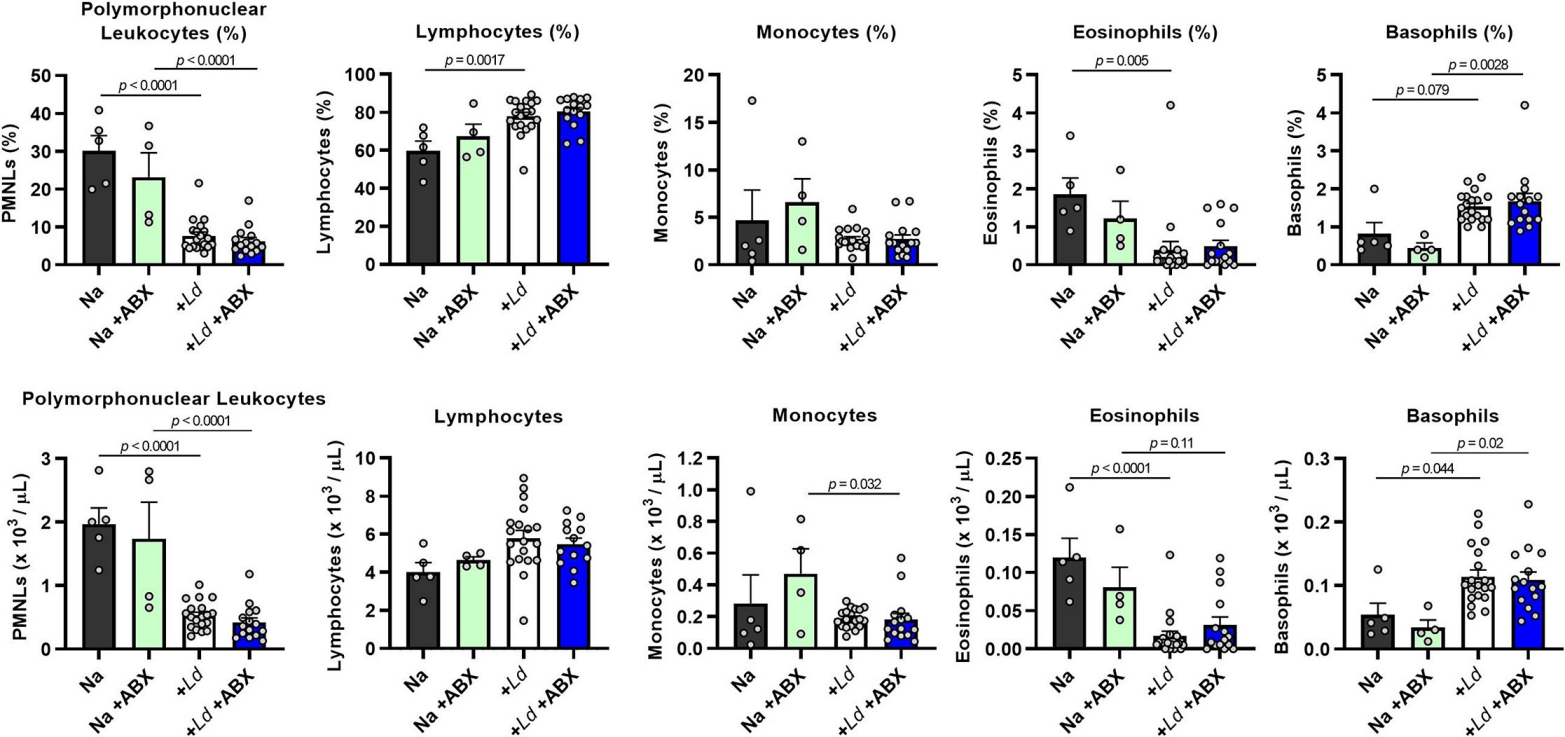
Leukocyte profiles of dysbiotic hamsters infected with *L*. *donovani*. Hamsters were maintained on drinking water with or without antibiotic cocktail (ABX) and infected with *L*. *donovani* (n = 15–19) and compared with naïve (Na) controls (n = 4–5). Data show individual component assays from complete blood counts at 12 weeks post-infection. Groups were compared by one way ANOVA and differences at *p* < 0.05 are indicated.

**Fig 6:**
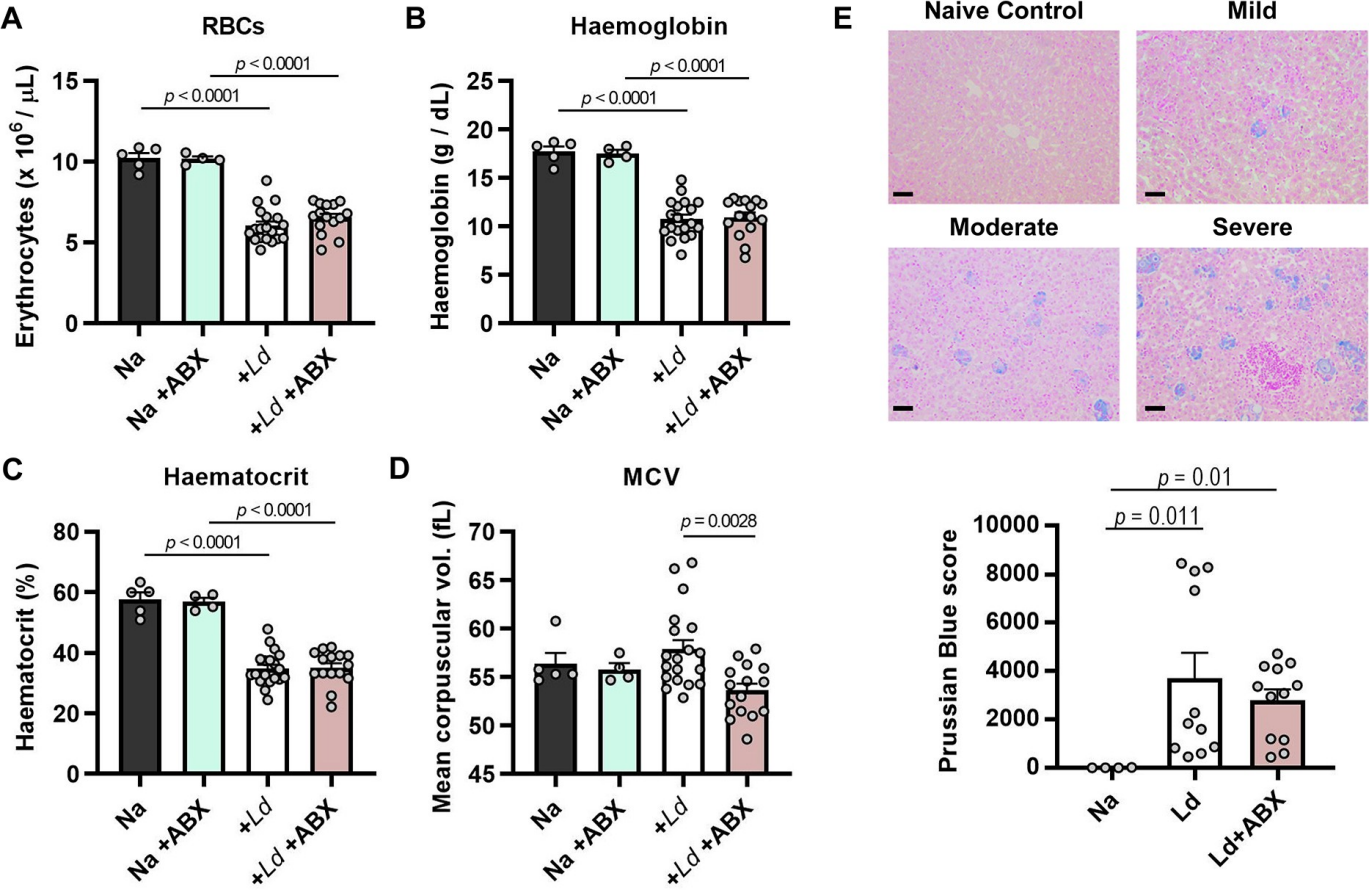
Anaemia and iron sequestration in dysbiotic hamsters infected with *L*. *donovani*. Hamsters were maintained on drinking water with or without antibiotic cocktail (ABX) and infected with *L*. *donovani* (n = 15–19) and compared with naïve (Na) controls (n = 4–5). The erythrocytic compartment was analysed with respect to total red cell count (A), haemoglobin levels (B), haematocrit (C) and mean corpuscular volume (D). (E) Representative images and quantification of ferric iron content in liver sections stained with Prussian blue (n = 4–12), magnification 200X, scale bars 50 μm. All data are from 12 weeks post-infection, with mean +SEM and individual hamster data points shown. Groups were compared by one way ANOVA (A-D) or Kruskal-Wallis test (E) and differences at *p* < 0.05 are indicated.

Haematological disorders and parasite persistence in bone marrow are common features of human VL [26, 27] and we reasoned these could be relevant to disease amelioration in ABX-treated hamsters. We detected high levels of *L*. *donovani* infection in bone marrow in hamsters at 12 weeks p.i., but parasite loads were not significantly different in ABX-treated animals compared to controls and they did not correlate with disease progression (S3 Fig). We next analysed leukocyte subsets in peripheral blood (Fig 5) and found that the frequency and abundance of polymorphonuclear cells and eosinophils were significantly reduced in infected hamsters, while basophil levels were significantly increased. Leukocyte profiles were not significantly altered by ABX-treatment at this time point. Analysis of the red blood cell compartment revealed pronounced anaemia in both ABX-treated and untreated, infected hamsters at 12 weeks p.i., evidenced by significantly reduced erythrocyte numbers, haemoglobin levels and packed cell volume (Fig 6A–6C). ABX-treated hamsters had a significantly lower mean corpuscular volume than untreated controls (Fig 6D) possibly reflecting lower reticulocyte production. Consistent with the observed anaemia, we found clear histological evidence of iron sequestration in these hamsters’ livers (Fig 6E). The amount of sequestered iron showed high intra-group variability and although the means were not significantly different between ABX-treated and untreated controls, the most severe cases (Prussian blue scores > 6000) were only seen in control hamsters.

### Immune response profiles

**Fig 7:**
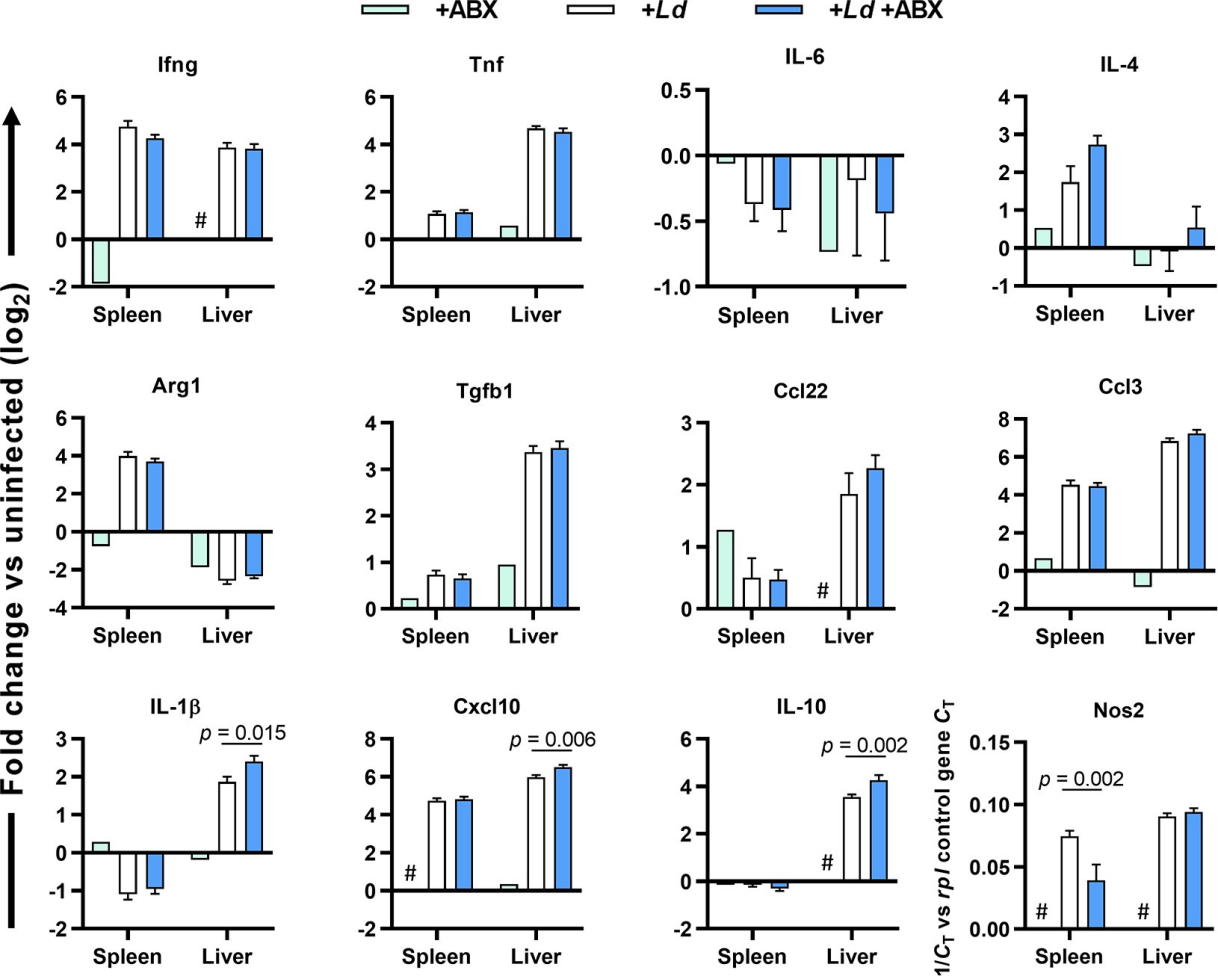
Gene expression analysis in dysbiotic hamsters infected with *L*. *donovani*. Hamsters were maintained on drinking water with or without antibiotic cocktail (ABX) and infected with *L*. *donovani* (n = 6) or kept naïve (n = 1). At 12 weeks post-infection, RNA transcript abundance for the indicated genes in spleen and liver tissue was measured by RT-qPCR with normalisation to *Rpl18*. Data are mean +SEM log_2_-transformed fold change in gene expression compared to untreated, uninfected control hamsters (n = 4). # indicates transcript abundance was below detection limit. Differences are indicated when *p* < 0.05 between groups in t-test.

Parasite-driven immune responses in chronic *L*. *donovani* infections are key mediators of tissue damage [28] and likely have roles in driving cachexia in progressive VL [24]. We therefore performed expression analysis of spleen and liver tissue at 12 weeks p.i. for a set of genes suspected to be important in hamster VL pathogenesis (Fig 7). Infected hamsters in both groups displayed strong upregulation of multiple pro-inflammatory mediators as well as counter-regulatory factors including TGF-β and IL-10, consistent with chronic infection in these tissues. Analysis of samples from an uninfected, ABX-treated control showed these changes were specifically induced by the infection (Fig 7). Four genes were differentially expressed between ABX-treated and untreated infected hamsters: (i) decreased iNOS in the spleen; (ii) increased IL-1β in the liver; (iii) increased CXCL10 (IP-10) in the liver; and (iv) increased IL-10 in the liver. These differences in gene expression suggest a subtle but significant shift in the host response in ABX-treated hamsters. Whether or not these differences contribute to their survival advantage is not clear.

### Parasitism of the GI tract and bacterial translocation in progressive hamster VL

**Fig 8:**
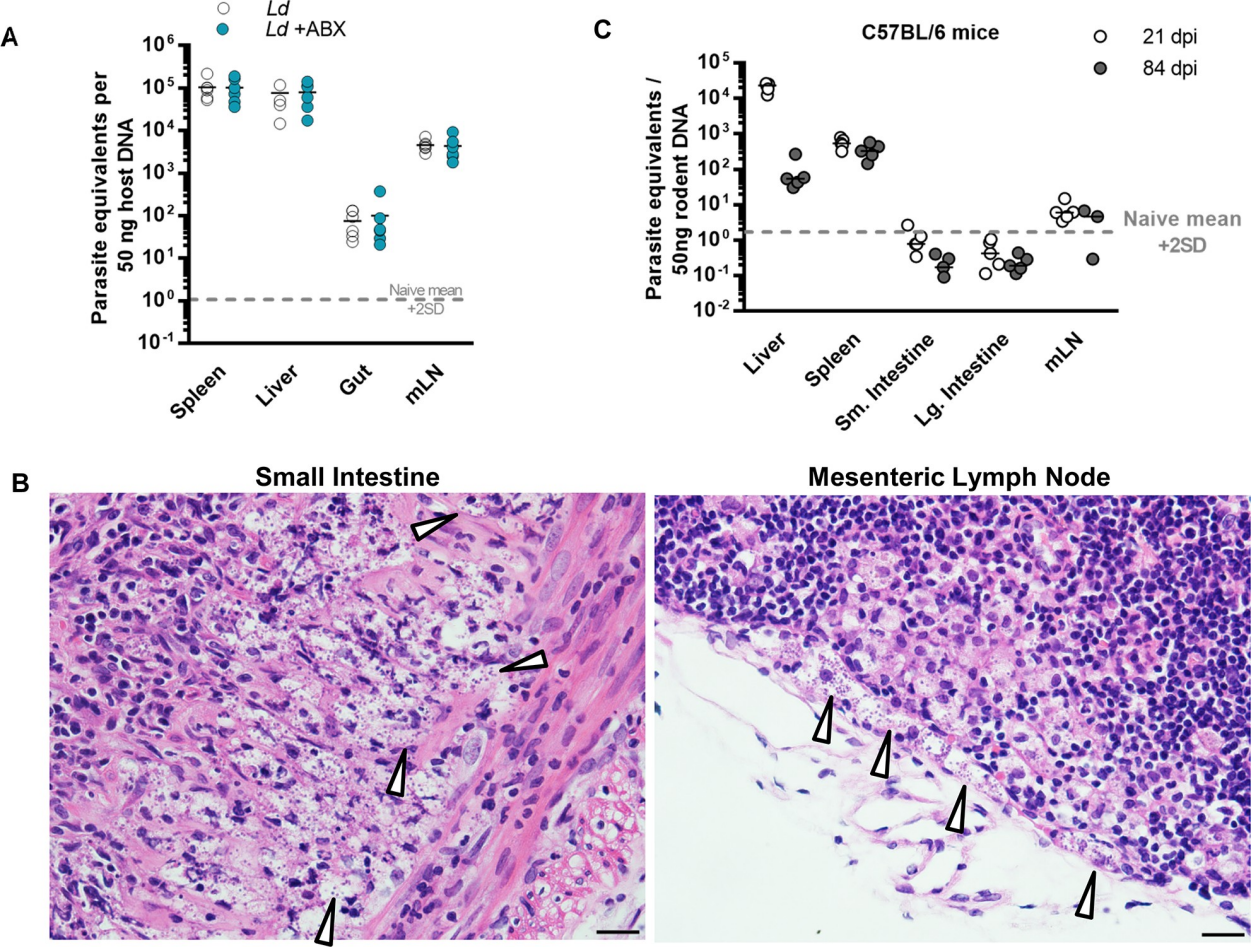
The GI tract is a site of chronic *L*. *donovani* infection in hamsters but not mice. (A, C) qPCR analysis of *L*. *donovani* parasite loads in tissues from hamsters (A) and mice (C). Parallel reactions for a parasite-specific target (kDNA minicircle) and a host-specific gene (*Actbl2*) were performed on DNA extracted from the indicated tissues at 84 days post-infection (dpi) for hamsters (n = 6 per group) and 21 and 84 dpi for mice (n = 3–5 per group). Lines show medians and dashed line shows the detection limit (mean + 2 standard deviations of uninfected control samples, n = 4). (B) Histological sections for hamster small intestine (left, centre) and mesenteric lymph node (right) stained with haematoxylin and eosin; arrows indicate amastigote forms of *L*. *donovani;* magnification 400X, scale bars 20 μm.

Our analyses of canonical *L*. *donovani* target tissues (spleen, liver, bone marrow) did not provide a clear explanation for the survival advantage seen in ABX-treated hamsters. The GI tract has previously been reported as a site of *L*. *donovani* infection in humans and dogs but is rarely considered in experimental studies of VL in rodents. We hypothesized that naturally abundant intestinal myeloid cell populations might support *L*. *donovani* infection and that this may be relevant for the mechanism of protection conferred by oral antibiotics. In support of this, a *L*. *donovani*-specific qPCR assay [29] allowed us to consistently detect parasite DNA in intestinal tissues and gut-draining lymph nodes from infected hamsters (Fig 8A). Intracellular amastigote forms were readily identifiable in histological sections and were particularly prevalent in the submucosa of the small intestine and the subcapsular sinus of the lymph node (Fig 8B). Parasite loads in these sites were equivalent in infected hamsters given ABX and control water (Fig 8A). Intestinal parasitism was not a feature of acute or chronic infections in mice, although very low amounts of parasite DNA were detected in the gut-draining lymph nodes (Fig 8C). This indicates that intestinal infection is a specific feature of progressive VL.

**Fig 9:**
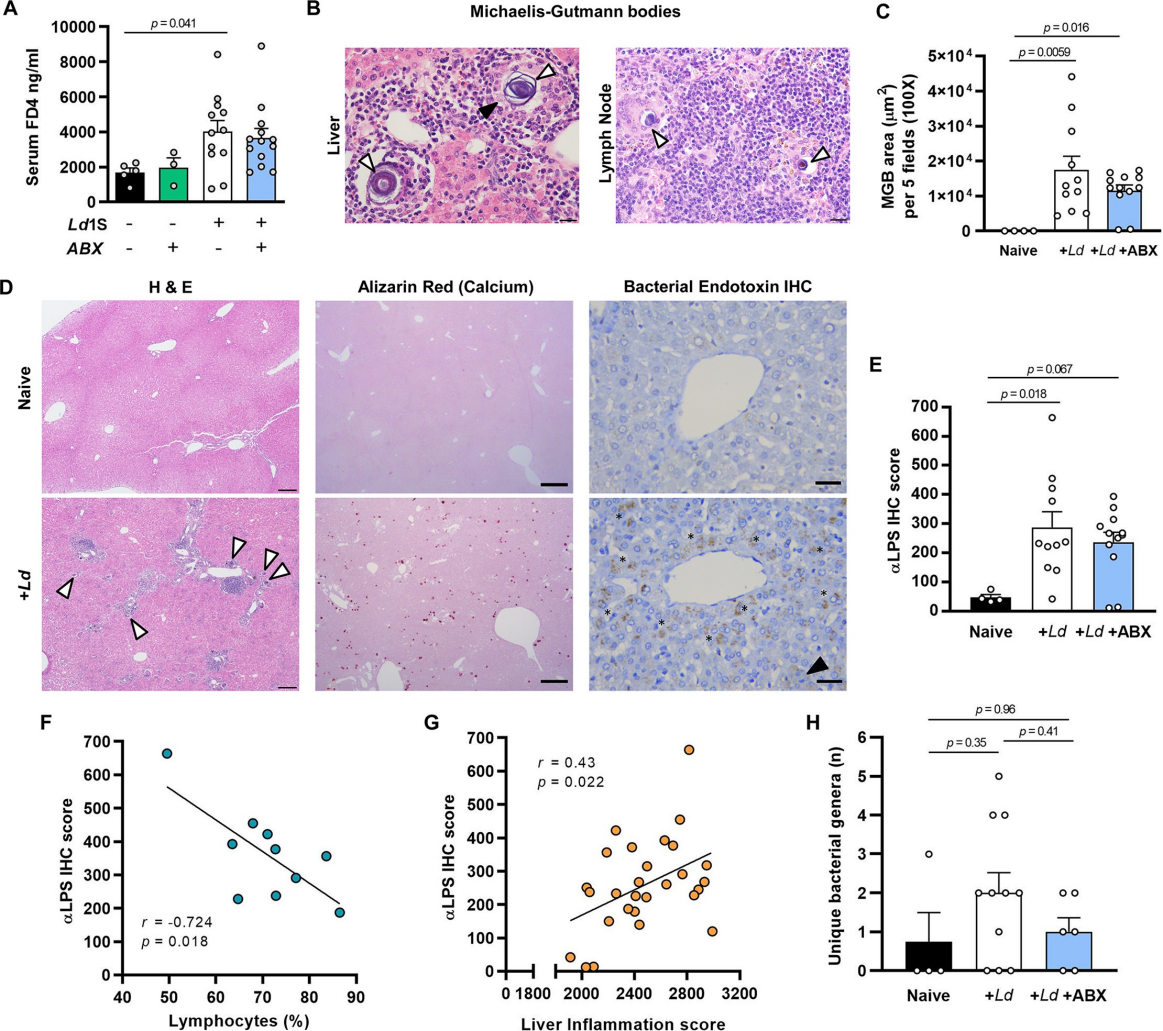
Evidence of bacterial co-infection in hamster visceral leishmaniasis. (A) Naïve or *L*. *donovani* (*Ld*) infected hamsters maintained on drinking water with or without antibiotic cocktail (ABX) were fed fluorescently-labelled 4 kDa dextran at 12 weeks post-infection (wpi). After 4 hours the intensity of fluorescence in serum samples was measured as an indicator of intestinal barrier leakiness. (B) Histological sections stained with haematoxylin and eosin showing presence of Michaelis-Gutmann bodies (MGBs, white arrows) in *L*. *donovani* infected liver and duodenal lymph node tissue, black arrow indicates intracellular amastigotes. (C) Quantification of MGB abundance at 12 wpi. (D) Histological liver sections stained with haematoxylin and eosin (left), alizarin red for calcium detection (centre) and immunohistochemical (IHC) detection of bacterial endotoxin (right), white arrows indicated MGBs, black arrow indicates intracellular amastigotes, asterisks indicate anti-endotoxin IgG-reactive areas. Image magnifications and scale bars are 400X and 20 μm (C), 12.5X and 1 mm (D, H&E, upper), 40X and 200 μm (D, H&E, upper) 100X and 500 μm (alizarin), 400X and 30 μm (IHC). (E) Quantification of anti-endotoxin IgG-reactive areas at 12 wpi. (F,G) Pairwise comparison and Pearson correlation tests of liver endotoxin content and peripheral blood lymphocyte frequency (F, n = 10) and liver inflammation scores (G, n = 28). (H) Quantification of unique bacterial genera present in liver tissue assessed by 16S rDNA sequencing. Data are for male hamsters. Data on charts (A,C,E,H) are means +SEM, compared by one-way ANOVA, n = 3–12.

Chronic parasitism of the hamster GI tract by *L*. *donovani* led us to question whether its barrier function might be compromised, potentially leading to bacterial translocation. To test this, fluorescently-labelled 4 kD dextran was fed to hamsters at 12 weeks post-infection. After four hours a significant increase in fluorescence was detected in serum from infected hamsters (Fig 9A). Dextran translocation was not significantly different between the ABX-treated and untreated animals (Fig 9A), consistent with their similar intestinal parasite loads (Fig 8A). This suggested that translocation of bacteria from the gut could be a feature of hamster VL. Additional evidence for this was found in histological sections of the liver and lymph nodes associated with the duodenum, in which Michaelis-Gutmann bodies (MGBs) were found (Fig 9B and 9C). These structures are pathognomonic of malakoplakia, a rare condition most often seen in urogenital tract infections caused by gram-negative bacteria [30]. It is thought that MGBs result from mineralisation of macrophages that are defective in their bactericidal activity. We confirmed the identity of MGBs by staining sections for calcium using alizarin red and found they were present at a very high frequency in infected hamster livers (Fig 9C and 9D). These were less abundant on average in hamsters given ABX compared to untreated controls, though not significantly so (Fig 9F). MGBs were a specific feature of progressive VL because they were absent from *L*. *donovani*-infected mice. These data led us to ask whether secondary bacterial infection could explain the presence of MGBs. Using immunohistochemical labelling for a conserved LPS epitope we detected significant levels of this bacterial cell wall component in *L*. *donovani*-infected livers (Fig 9C and 9E). Our hamsters were housed under SPF conditions and LPS was predominantly localised in the areas surrounding blood vessels, so we reasoned that the intestinal microbiota was the source of secondary bacterial infection. The amount of LPS was negatively correlated with lymphocyte frequency (Fig 9F) and positively correlated with liver inflammation (Fig 9G) leading us to conclude that the staining likely reflected the presence of live bacteria rather than just LPS. The level of anti-LPS staining was slightly lower on average in ABX-treated animals’ livers but this difference was not significant (Fig 9G). This indicates that bacteria resistant to the ABX treatment were able to translocate to the liver to a similar extent to that seen in *Ld*-infected animals given control water. We readily isolated live bacteria from liver tissue homogenates under aerobic *in vitro* growth conditions (Table 1, S4 Fig) Typing by 16S rRNA amplicon sequencing revealed the presence of nine bacterial genera in hamsters from the untreated, infected group compared to five genera for the ABX-treated group. *Paenibacillus* and *Staphylococcus* were suspected as contaminants given the ubiquity of the former, even in naïve untreated hamsters and the abundance of the latter in skin and environmental control swab samples (S4 Fig). On average, fewer unique genera were recovered from individual ABX-treated hamsters than untreated controls, although this was not statistically significant (Fig 9H). However, by analysing the frequency of each bacterial genus (Table 1) we found a significant reduction in *Rodentibacter* (Gram-negative, formerly *Pasteurella* [31]) in ABX-treated animals (7 of 11 untreated vs 0 of 6 ABX-treated, *p* = 0.035). *Streptococcus* was also markedly less common, though not to a significant level (7 of 11 untreated vs 1 of 6 ABX-treated, *p* = 0.13). Interestingly, given the VL anaemia phenotype, haemolysis of blood agar was observed for five of the hamster livers testing positive for *Streptococcus*.

**Table 1:** Frequency of live bacterial genera isolated from hamster liver homogenates.

Genus	Naïve		*Ld*1S Infected				
	Not Treated (n = 4)		Not Treated (n = 11)		ABX treated (n = 6)		
	Frequency	95% CI	Frequency	95% CI	Frequency	95% CI	*p*
***Paenibacillus***	1.000	(1–0.51)	0.727	(0.902–0.434)	0.833	(0.991–0.436)	>0.999
***Streptococcus***	0.000	(0.489–0)	0.636	(0.848–0.353)	0.167	(0.563–0.008)	0.131
***Granulicatella***	0.250	(0.699–0.012)	0.273	(0.565–0.097)	0.000	(0.39–0)	0.515
***Bacillus***	0.000	(0.489–0)	0.091	(0.377–0.004)	0.167	(0.563–0.008)	>0.999
***Rodentibacter***	0.250	(0.699–0.012)	0.636	(0.848–0.353)	0.000	(0.39–0)	**0.035**
***Lactobacillus***	0.000	(0.489–0)	0.091	(0.646–0.151)	0.333	(0.39–0)	0.515
***Staphylococcus***	0.250	(0.699–0.012)	0.364	(0.646–0.151)	0.000	(0.39–0)	0.237
***Actinomyces***	0.000	(0.489–0)	0.091	(0.377–0.004)	0.000	(0.39–0)	>0.999
***Escherichia***	0.250	(0.699–0.012)	0.182	(0.476–0.032)	0.000	(0.39–0)	0.515
***Klebsiella***	0.000	(0.489–0)	0.000	(0.258–0)	0.333	(0.7–0.059)	0.110
***Morganella***	0.000	(0.489–0)	0.000	(0.258–0)	0.000	(0.39–0)	>0.999
***Proteus***	0.000	(0.489–0)	0.000	(0.258–0)	0.000	(0.39–0)	>0.999

Our ABX cocktail contained ampicillin and neomycin, which may have had systemic effects after absorption. To test more directly whether the intestinal microbiota are the source of systemic bacterial pathogens we used oral monotherapy with either vancomycin or rifaximin, whose effects are confined to the intestine. There was no evidence of protection from VL progression in either monotherapy group: weight profiles, hepatosplenomegaly severity, parasite loads and anaemia were equivalent to untreated controls (S5 Fig). Live bacteria were still recovered from these hamsters’ livers. *Streptococcus* was significantly less frequent in the vancomycin-treated group but otherwise 16S rRNA sequence profiles were similar to the untreated controls (S2 Table). Therefore, either the bacterial species relevant to the protection seen in ABX-treated animals were insensitive to the monotherapies or they do not originate from the gut, or both.

In summary, these data show that intestinal infection with *L*. *donovani* is a feature of severe progressive VL and this is associated with barrier permeability, translocation of diverse bacteria to the liver and the formation of calcified MGBs. Hamsters treated with a combination of ampicillin, neomycin and vancomycin were significantly less likely to harbour systemic *Rodentibacter* than untreated controls, indicating that these bacteria might contribute to the fatal progression of VL in the hamster model.

## Discussion

A largely neglected area of investigation pertaining to the variable clinical outcomes in individuals infected with *L*. *donovani* is the influence of the host’s microbiota in preventing or promoting VL pathogenesis. We have used the hamster model of VL, which mimics some key clinicopathological features of active human disease, to explore the effect of intestinal dysbiosis on the course of disease. *L*. *donovani*-infected hamsters that were maintained on broad spectrum antibiotics in their drinking water had delayed onset of weight loss, reduced hepatosplenomegaly, and significantly improved nine-month survival compared to untreated controls. Strikingly, the protection in the dysbiotic hamsters was not associated with lower visceral parasite burdens. Progressive disease was associated with intestinal parasitism, compromised barrier function, and evidence for translocation of live bacteria to the liver. The findings are the first to directly link the host microbiota to clinical outcome and to show the benefits of antimicrobial therapy in the treatment of VL.

Our findings extend the evidence base that hamsters are a suitable model of symptomatic human VL. Specifically, we draw attention to the potential importance of a gut-liver axis in disease pathogenesis. The liver is well established as a site of non-resolving *L*. *donovani* infection in hamsters and humans, in contrast to the largely asymptomatic mouse model. Features of hepatic pathology included severe chronic granulomatous inflammation, malakoplakia and iron sequestration, which were all absent from the mouse model. Our data unequivocally show that parasitism of the intestine is a feature of hamster and not murine VL. This site is rarely studied in rodent models although infections have previously been described for *L*. *braziliensis* [32] and *L*. *donovani* [33]. Intestinal parasitism and symptoms are common in canine VL caused by *L*. *infantum* [34, 35]. Some data indicate that the gut is an important site of parasite persistence in human VL. Anorexia, abdominal pain and weight loss are all symptoms of acute infection with *L*. *donovani; s*ymptomatic gastrointestinal leishmaniasis is relatively common in immunosuppressed and HIV co-infected patients [36], and there are occasional reports of discovery of *Leishmania* parasites in the GI tracts of immunocompetent individuals [37, 38]. Against this background, our data suggest that intestinal parasitism is likely to be more common and more important to VL outcomes than previously thought.

Secondary bacterial infections in VL have been attributed to environmental and nosocomial sources or to underlying infections such as tuberculosis [39–41]. Our findings for hamsters kept in a highly controlled, pathogen-free environment raise the possibility that a patient’s endogenous microbiota also needs to be considered. Our data indicated that *Rodentibacter* are prime candidates contributing to hamster VL progression. This genus includes rodent pathobiont species that can cause opportunistic infections, septicaemia and severe pneumonia [42, 43]. *L*. *donovani* infection of the intestinal mucosa may be directly related to the increased gut barrier permeability observed during progressive disease, similar to that produced by enteropathogens and other systemic infections (reviewed in [44]). We obtained direct evidence for compromised barrier integrity in VL from the elevated serum levels of orally administered fluorescently-labelled 4 kDa dextran. The perivascular staining of LPS in the liver was also consistent with bacterial translocation from the gut, as was the presence of MGBs, which comprise calcified macrophages containing partially digested bacteria. The lack of impact by non-absorbed antibiotics indicates that the protection conferred by ABX treatment is probably mediated by ampicillin and/or neomycin activity against bacteria in sites other than the intestinal lumen. This could be either via redistribution of the drug(s) after absorption or via direct action in the naso/oropharynx. *Rodentibacter* can be found in the intestine, but are more commonly known to colonise the upper respiratory tract and oral cavity [45, 46]. Human mucosal leishmaniasis caused by *L*. *dononvani* is well known, particularly in Sudan from where the Ld1S strain originates [47–51]. Future experimental studies should therefore test whether *L*. *donovani* persists in the hamster respiratory tract and mucocutaneous tissues, where it may promote translocation of bacterial pathobionts such as *Rodentibacter*.

While these are the first observations of their kind in an animal model of VL, a series of clinical findings support a role for bacterial translocation in human VL [52, 53]. In these studies of Brazilian VL patients, including those with HIV co-infection, augmented levels of plasmatic LPS were observed that positively correlated with upregulated expression of pro-inflammatory cytokines and cellular activation markers, responses that over time were argued to result in T cell depletion and exhaustion (reviewed in [54]). A strikingly pro-inflammatory environment has been described in livers and spleens of *L*. *donovani* infected hamsters by transcriptional profiling [13, 55]. Our results are largely consistent with those findings, with transcripts for selected inflammatory mediators, e.g. IFNγ, TNFα, IL-1β, CXCL10, showing strong upregulation, particularly in the liver, though upregulation of counter-regulatory cytokines, eg, IL-10, TGFβ, was also observed. The ABX-treatment group did show significantly increased levels of IL-10 in the liver, which could have a tissue-protective effect. On the whole though, differences in immune response profiles were moderate and possibly too selective to meaningfully attribute their survival benefit to attenuation of a bacteria-driven cytokine storm within the spleen or liver.

The hamster is an outbred animal model and we observed high intra-group variability in many of our data sets. Consequently, our sample sizes had limited statistical power to detect moderate effects. For example, many of our assays were carried out at 12 weeks post-infection and at this time point there was a wide range of progression rates (in terms of weight loss) within both experimental groups. With the possible exception of reduced hepatosplenomegaly, our analyses of parasite loads, pathology and immune responses in multiple tissues at this time point failed to identify specific factors that were sufficiently different in ABX-treated hamsters to explain their slow progression phenotype. Conversely, our analyses on humane end-point samples, taken from animals that had reached the same stage of progression (over largely variable time periods), were of limited value for establishing an upstream mechanism. The main protective effect in treated hamsters could be attributed to the absence of rapid progression, which occurred in approximately half of untreated controls. Therefore, analysis of additional time points with larger animal cohorts could aid future investigation of this phenomenon. Other factors that were not part of our study could also be relevant. For example, long-term antibiotic-induced dysbiosis may have nutritional consequences such as caloric restriction or altered availability of micro-nutrients. However, this seems an unlikely explanation for the observed protection because malnutrition is well-established as a risk factor in human VL [10]. Furthermore, although we were able to type some bacterial genera in the VL affected liver, it may be that critical species were not viable under the culture conditions that we used. Our method of monitoring the effect of antibiotics using 16S rRNA on faecal pellets was faeces may not accurately represent the effect of antibiotics on the small intestinal flora. Metagenomic sequencing strategies may reveal additional candidate bacterial species involved in accelerating VL progression.

Bacterial sepsis is a leading cause of death in VL but appropriate antibiotic treatment may not be initiated until a culture-positive diagnostic test result is obtained [40, 56, 57]. There are reports that antibiotic prophylaxis is already used in some endemic areas as an adjunct to anti-parasitic drug therapy for VL [58]. Our data provide support for efforts to obtain empirical evidence that this approach has clinical benefit for VL patients.

## Materials and methods

### Ethics statement

All experiments were performed in strict accordance with the recommendations in the Guide for the Care and Use of Laboratory Animals of the NIH. The protocols were approved by the Animal Care and Use Committee of the NIAID (protocol LPD 68E). Animals were euthanised by CO_2_ inhalation.

### Animals

C57BL/6 mice were bred in-house and Golden Syrian hamsters were bought from Harlan Laboratories (Indianapolis, IN). They were maintained under specific pathogen-free conditions in in the animal care facility of the National Institute of Allergy and Infectious Diseases (NIAID), National Institutes of Health (NIH). They experienced a 12 hour light/dark cycle and had access to food and water ad libitum. Female mice were used at age 8–10 weeks. Female and male hamsters were used at age 4–6 weeks. The primary humane end point was loss of 20% of body weight compared to the start of infections. Secondary humane end points included sustained lethargy or absence of feeding or drinking behaviour.

### Antibiotics

To induce intestinal dysbiosis, animals were given ad libitum water containing ampicillin (1 mg/ml), neomycin (1 mg/ml) and vancomycin (0.5 mg/ml) (“ABX”), or in some cases vancomycin only. In some experiments hamsters were given rifaximin (100 mg/kg) in Ora-Blend vehicle (Perrigo) p.o. three times per week. In some experiments with C57BL/6 mice, metronidazole (1 mg/ml) and sucralose (3 mg/ml) was included in the ABX cocktail. Antibiotic-containing water was replaced weekly. Treatment was initiated 4 weeks prior to *L*. *donovani* infection and sustained throughout the experiments.

### Parasites and infections

In vitro promastigote cultures of the *L*. *donovani* strain 1S (MHOM/SD/00/1S-2D) were established from frozen stocks of tissue amastigotes at 26°C in medium 199 supplemented with 20% heat-inactivated fetal calf serum, 100 U/mL penicillin, 100 mg/mL streptomycin, 2 mM L-glutamine, 40 mM Hepes, 0.1 mM adenine (in 50 mM Hepes), 5 mg/mL hemin (in 50% triethanolamine), and 1 mg/mL 6-biotin (M199/C). Minimally passaged promastigotes were seeded at 5x10^5^ /ml and 5–6 days later metacyclic promastigotes were purified by centrifugation through a Ficoll gradient [59]. Hamsters were infected by intracardiac injection while under gaseous anaesthesia (2% isoflurane in oxygen) with 3 × 10^7^ metacyclic promastigotes. Mice were infected by intravenous injection with 1x10^7^ metacyclic promastigotes.

### Necropsies

Animals were euthanised by CO_2_ inhalation. This was followed by cardiac exsanguination and trans-cardial perfusion with 10 ml (mice) or 20 ml (hamsters) PBS. Spleens, livers, caeca, small and large intestines, mesenteric lymph nodes and femurs were collected as required. Tissue fragments were weighed and either snap-frozen on dry ice, fixed in 10% neutral buffered formalin or transferred to ice cold M199/C media.

### Parasite loads—*ex vivo* titrations

Parasite loads in livers, spleens and bone marrow were determined using an *ex vivo* limiting dilution assay [60]. Briefly, organs were dissociated by mashing through a 70 μm cell strainer and femurs were flushed with M199/C media, then cells were collected by centrifugation at 3000 x *g* for 15 mins. The cell pellet was washed in media, centrifuged again and then erythrocytes were lysed by incubation for 5 mins in ACK buffer (Lonza, USA). Cells were washed with excess PBS, harvested by centrifugation and re-suspended in M199/C. Duplicate two-fold dilution series to extinction were made in M199/C in 96 well culture plates (Nunc). The culture plates were sealed with parafilm, and incubated at 27°C. After 21 days, all wells were examined microscopically and scored for *L*. *donovani* promastigote growth. The number of parasites per organ was extrapolated from the number of parasite positive wells.

### Quantitative PCR for parasite loads

For DNA extraction, frozen tissue samples were thawed and immediately homogenized in at least 400 μl lysis buffer (4M urea, 200 mM Tris, 20 mM NaCl, 200 mM EDTA, pH 7.4) per 50 mg tissue using a Precellys 24 instrument (Bertin). The tissue suspension was then incubated overnight at 37°C with 0.6 mg proteinase K in a shaking incubator. DNA was extracted using the High Pure PCR Template Preparation Kit (Roche) according to the manufacturer’s instructions. Real-time PCR reactions were prepared using the SensiFAST SYBR Kit (Bioline) and run on an ABI Prism 7900HT or QuantStudio6 instrument (Applied Biosystems) using an annealing temperature of 59°C. Reactions were run in duplicate and contained 10–100 ng DNA and 0.2 μM of each primer. Separate reactions were performed to quantify *L*. *donovani* using primers targeting kDNA minicircles and mouse or hamster DNA using conserved primers targeting the *actbl2* gene. Primer sequences were as follows: kDNA-F CTTTTCTGGTCCTCCGGGTAGG, kDNA-R CCACCCGGCCCTATTTTACACCAA [29], ACTBL2-F AAGGACTGTTATGTGGGAG and ACTBL2-R GTGTGGTACCAGATCTTCTC. Measurements of *L*. *donovani* and mouse/hamster DNA content were established using standard curves.

### Quantitative PCR for bacterial 16S rDNA

DNA was extracted from frozen faecal pellets using the QIAamp DNA Stool Mini Kit according to the manufacturer’s instructions with modification whereby pellets were incubated in lysis buffer overnight in a shaking incubator. Real-time PCR reactions were prepared using the SensiFAST SYBR Kit (Bioline) and run on an ABI Prism 7900HT or QuantStudio6 instrument (Applied Biosystems) using an annealing temperature of 63°C. Reactions were run in duplicate and contained 10–100 ng DNA and 0.2 μM of primers F340 (ACTCCTACGGGAGGCAGCAGT) and R514 (ATTACCGCGGCTGCTGGC) [61]. A full length 16S rDNA sequence was PCR amplified and cloned into pCR2.1 using the TA Cloning kit (Invitrogen). A dilution series of the resulting plasmid, pCR2.1-16S, was used as a standard curve to establish 16S rDNA copy number in faecal samples.

### Quantitative PCR for immune response gene expression

Frozen tissue samples were thawed and immediately homogenized in at least 400 μl Tri-reagent (SigmaAldrich) per 50 mg tissue using a Precellys 24 instrument (Bertin) then mixed with 0.2 volumes of chloroform. The aqueous phase was separated by centrifugation at 12000 x *g* and then processed using the RNeasy kit (Qiagen) with on-column DNAse I treatment. cDNA synthesis reactions were prepared using 1 μg RNA and the SuperScript III first-strand synthesis system for reverse transcription (Invitrogen). Real-time PCR reactions were prepared using the SensiFAST SYBR Kit (Bioline) and run on a QuantStudio6 instrument (Applied Biosystems) using an annealing temperature of 60°C. The relative expression level of each gene was determined by the comparative *C*_T_ method [62] using *rpl18* as the endogenous control gene. Primer sequences are given in S1 Table.

### Hematology and serology

Automated complete blood counts were conducted by the NIH Department of Laboratory Medicine hematology service. Leishmania-specific IgG serum titres were determined by ELISA using total soluble leishmania antigen (SLA). In brief, Immunol 4X HB plates (ThermoFisher) were coated with 20μg/ml SLA in carbonate-bicarbonate coating buffer overnight. Plates were blocked with 4% BSA before washing and adding diluted sera. After washing, an anti-hamster IgG ALP (Sigma Aldrich) secondary antibody in conjunction with pNPP were used to detect bound anti-*Leishmania* antibody. To test intestinal barrier permeability, hamsters were fasted for 12 hours then orally dosed with 50 mg FITC-labelled 4 kDa dextran (Sigma). After four hours blood was collected, allowed to clot and serum was then isolated. Fluorescence in serum samples and a standard curve was measured in a plate reader (Molecular Devices) with filters for excitation at 485 nm and emission at 528 nm.

### Histopathology and immunohistochemistry

Tissue samples were fixed in neutral buffered 10% formalin for 24–72 hours, then dehydrated, cleared and embedded in paraffin. For general histopathological assessment, performed by a board-certified veterinary pathologist, three micron sections were stained with haematoxylin and eosin. An inflammation index was derived by quantifying the number of nuclei in these stained sections. For calcium detection sections were stained with alizarin red for 2 mins, washed in running water then dehydrated in acetone, followed by 50% acetone in xylene (vol/vol). For iron detection, sections were treated with 20% hydrochloric acid and 10% potassium ferrocyanide for 20 mins and then counterstained with nuclear fast red for 3 minutes. Bacterial endotoxin content was quantified using immunohistochemistry. For this, sections were subjected to heat-induced epitope retrieval by incubation in 10 mM sodium citrate, 0.05% Tween20 for 30 mins, then cooled and rinsed in distilled water. Sections were blocked with 10% sheep serum and 1% BSA in TBS for 30 mins then incubated at 4°C overnight with 2 μg/ml monoclonal mouse anti-core LPS IgG (Hycult) and 1% BSA in TBS. Sections were then washed with 0.025% Triton X-100 in TBS and endogenous peroxidase activity was quenched with 3% H_2_O_2_ for 30mins. Bound primary antibody was labelled with 6 μg/ml biotinylated goat anti-mouse IgG and 1% BSA in TBS for 30 mins. Slides were then washed as previously, incubated with avidin-peroxidase (Vectalabs) for 30 mins, washed again and treated with DAB (Thermo) for 5 mins. Sections were counterstained with haematoxylin and mounted with DPX. For quantification of histological and immunohistochemical stains and features, images of five (alizarin) or ten (all other stains) fields of view were acquired using a camera (Leica DFC295) attached to a Leica DM3000 LED microscope. Images were digitized for histomorphometric analysis using the Leica Application Suite V4.5 software (Leica).

### Bacterial growth and 16S rDNA sequence typing

Liver tissue samples were stored in PBS on ice then transferred to a gentleMACS tube containing 3–10 ml PBS and homogenized using a gentleMACS dissociator (Miltenyi Biotec). Homogenate aliquots were spread onto three agar plates (Teknova) comprising (i) trypticase soy agar (TSA) + 0.1% w/v Tween-80, (ii) TSA + 5% v/v Sheep’s blood, and (iii) brain heart infusion agar. Plates were incubated for 24–48 hrs. If bacterial colonies were visible then a representative sample of the morphologies present was picked into nutrient-matched broth and incubated overnight with shaking at 225 rpm. DNA was extracted using the DNeasy Blood and Tissue kit (Qiagen) according to the manufacturer’s protocol, including the recommended pre-treatment steps for Gram-positive bacteria. 16S rDNA amplicons were generated by PCR using primers 27F (5’-AGAGTTTGATCMTGGCTCAG-3’) and 1492R: (5’- GGTTACCTTGTTACGACTT-3’) [63]. Consensus sequences for each amplicon were generated commercially (Eurofins genomics) and aligned against 16S rDNA reference sequences (NCBI) by BLAST to identify genera.

### Statistics

Individual animals were used as the unit of analysis, except where otherwise stated. No blinding or randomisation protocols were used. Statistical differences between groups were evaluated using the Mann-Whitney U test, one-way ANOVA with Sidak’s post-hoc correction for multiple comparisons, or the Kruskal-Wallis test with Dunn’s post-hoc correction. Pearson and Spearman log-rank correlation analyses were used to evaluate relationships between normally distributed and non-normally distributed variables respectively. Survival curves were compared by Gehan-Breslow-Wilcoxon test. Bacterial genus frequencies were compared using Fisher’s exact test. These tests were performed in GraphPad Prism v.7. Differences of *p* < 0.05 were considered significant.

## Supporting information

S1 Table RT-qPCR primers used in the study. (PDF)

S2 Table Frequency of live bacterial genera recovered from livers of *L*. *donovani*-infected hamsters treated with vancomycin or rifaximin. (PDF)

S1 Fig Comparison of VL related weight profiles for male and female hamsters. (TIF)

S2 Fig Pairwise comparisons of spleen and liver total parasite loads with VL progression. (TIF)

S3 Fig Bone marrow parasite loads and pairwise comparison with disease progression. Parasite loads were quantified by limiting dilution in vitro culture assay.
(TIF)

S4 Fig Analysis of 16S rRNA genetic diversity for live bacteria present in hamster liver tissue samples, skin swabs and environment. (TIF)

S5 Fig Evaluation of the impact of oral vancomycin or rifaximin monotherapy on hamster VL outcomes. (TIF)
