# Supplementary material for: Fatal progression of experimental visceral leishmaniasis is associated with intestinal parasitism and secondary infection by commensal bacteria, and is delayed by antibiotic prophylaxis
Source: PLoS Pathog. 2020 Apr 13;16(4):e1008456. doi: 10.1371/journal.ppat.1008456 (PMC7179947; doi:10.1371/journal.ppat.1008456)
Supplement: S1 Table — (PDF) [file ppat.1008456.s001.pdf]

Gene	Primer name	Primer sequence (5'-3')	Reference
**Rpl18**	Hm:RPL18-F	GTTTATGAGTCGCACTAACCG	Zivcec et al. 2011
	Hm:RPL18-R	TGTTCTCTCGGCCAGGAA	
**Ifng**	Hm:IFNg-F	AATATCTTGACGAACTGGCAAA	Espitia *et al*., 2014
	Hm:IFNg-R	CCTTCAAGGCTTCAAAGAGTTT	
**Tgfb1**	Hm:TGFb-F	GGCTACCACGCCAACTTCTG	Espitia *et al*., 2014
	Hm:TGFb-R	GAGGGCAAGGACCTTACTGTACTG	
**Tnf**	Hm:TNF-F	TGAGCCATCGTGCCAATG	Espitia *et al*., 2014
	Hm:TNF-R	AGCCCGTCTGCTGGTATCAC	
**Il10**	Hm:IL-10-F	GGTTGCCAAACCTTATCAGAAATG	Espitia *et al*., 2014
	Hm:IL-10-R	TTCACCTGTTCCACAGCCTTG	
**Il4**	Hm:IL-4-F	CCACGGAGAAAGACCTCATCTG	Zivcec et al. 2011
	Hm:IL-4-R	GGGTCACCTCATGTTGGAAATAAA	
**Il6**	Hm:IL-6-F	GGACAATGACTATGTGTTGTTAGAA	Ribeiro-Romao *et al*., 2016
	Hm:IL-6-R	AGGCAAATTTCCCAATTGTATCCAG	
**Nos2**	Hm:iNOS-F	TGAGCCACTGAGTTCTCCTAAGG	Osorio *et al*. 2012
	Hm:iNOS-R	TCCTATTTCAACTCCAAGATGTTCTG	
**Arg1**	Hm:ARG-F	ACCTATGTGTCATTTGGGTGGA	Osorio *et al*. 2012
	Hm:ARG-R	GCAGATATGCAGGGAGTCACC	
**Il1b**	Hm:IL-1b-F	GGCTGATGCTCCCATTCG	Zivcec et al. 2011
	Hm:IL-1b-R	CACGAGGCATTTCTGTTGTTCA	
**Ccl22**	Hm:CCL22-F	CGTGGCTCTCATCCTTCTTGC	Espitia *et al*., 2014
	Hm:CCL22-R	CAGATGCTGTCTTCCACGTTGG	
**Ccl3**	Hm:CCL3-F	CTCCTGCTGCTTCTTCTA	Matsui *et al*., 2011
	Hm:CCL3-R	TGGGTTCCTCACTGACTC	
**Cxcl10**	Hm:CXCL10-F	TGGAAATTATTCCTGCAAGTCA	Espitia *et al*., 2014
	Hm:CXCL10-R	GTGATCGGCTTCTCTCTGGT	

Zivcec M, Safronetz D, Haddock E, Feldmann H, Ebihara H. Validation of assays to monitor immune responses in the Syrian golden hamster (*Mesocricetus auratus*). *Journal of Immunological Methods*. 2011; 368(1):24-35.

Espitia CM, Saldarriaga OA, Travi BL, Osorio EY, Hernandez A, Band M, et al. Transcriptional profiling of the spleen in progressive visceral leishmaniasis reveals mixed expression of type 1 and type 2 cytokine-responsive genes. *BMC Immunology*. 2014; 15(1):38.

Osorio EY, Zhao W, Espitia C, Saldarriaga O, Hawel L, Byus CV, et al. Progressive Visceral Leishmaniasis Is Driven by Dominant Parasite-induced STAT6 Activation and STAT6-dependent Host Arginase 1 Expression. *PLOS Pathogens*. 2012; 8(1):e1002417.

Ribeiro-Romão RP, Saavedra AF, Da-Cruz AM, Pinto EF, Moreira OC. Development of real-time PCR assays for evaluation of immune response and parasite load in golden hamster (*Mesocricetus auratus*) infected by *Leishmania* (*Viannia*) *braziliensis*. *Parasites & Vectors*. 2016; 9(1):361.
