# Supplementary material for: Fatal progression of experimental visceral leishmaniasis is associated with intestinal parasitism and secondary infection by commensal bacteria, and is delayed by antibiotic prophylaxis
Source: PLoS Pathog. 2020 Apr 13;16(4):e1008456. doi: 10.1371/journal.ppat.1008456 (PMC7179947; doi:10.1371/journal.ppat.1008456)
Supplement: S2 Table — (PDF) [file ppat.1008456.s002.pdf]

Table S2. Frequency of live bacterial genera recovered from livers of *L. donovani*-infected hamsters treated with vancomycin or rifaximin

Genus	*Ld*1S Infected					
	Vancomycin treated (n = 6)			Rifaximin treated (n = 8)		
	Frequency	95% CI	*p*	Frequency	95% CI	*p*
Paenibacillus	0.333	(0.7 - 0.059)	0.162	0.375	(0.694 - 0.136)	0.181
Streptococcus	0.000	(0.39 - 0)	**0.035**	0.375	(0.694 - 0.136)	0.370
Granulicatella	0.000	(0.39 - 0)	0.515	0.000	(0.324 - 0)	0.228
Bacillus	0.000	(0.39 - 0)	>0.999	0.000	(0.324 - 0)	>0.999
Rodentibacter	0.500	(0.812 - 0.187)	0.644	0.375	(0.694 - 0.136)	0.370
Lactobacillus	0.500	(0.563 - 0.008)	0.099	0.125	(0.47 - 0.006)	>0.999
Staphylococcus	0.167	(0.563 - 0.008)	0.600	0.125	(0.47 - 0.006)	0.338
Actinomyces	0.000	(0.39 - 0)	>0.999	0.125	(0.47 - 0.006)	>0.999
Escherichia	0.167	(0.563 - 0.008)	>0.999	0.125	(0.47 - 0.006)	>0.999
Klebsiella	0.167	(0.563 - 0.008)	0.353	0.000	(0.324 - 0)	>0.999
Morganella	0.167	(0.563 - 0.008)	0.353	0.000	(0.324 - 0)	>0.999
Proteus	0.000	(0.39 - 0)	>0.999	0.125	(0.47 - 0.006)	0.421
