# Supplementary figures and images for: Fatal progression of experimental visceral leishmaniasis is associated with intestinal parasitism and secondary infection by commensal bacteria, and is delayed by antibiotic prophylaxis
Source: PLoS Pathog. 2020 Apr 13;16(4):e1008456. doi: 10.1371/journal.ppat.1008456 (PMC7179947; doi:10.1371/journal.ppat.1008456)

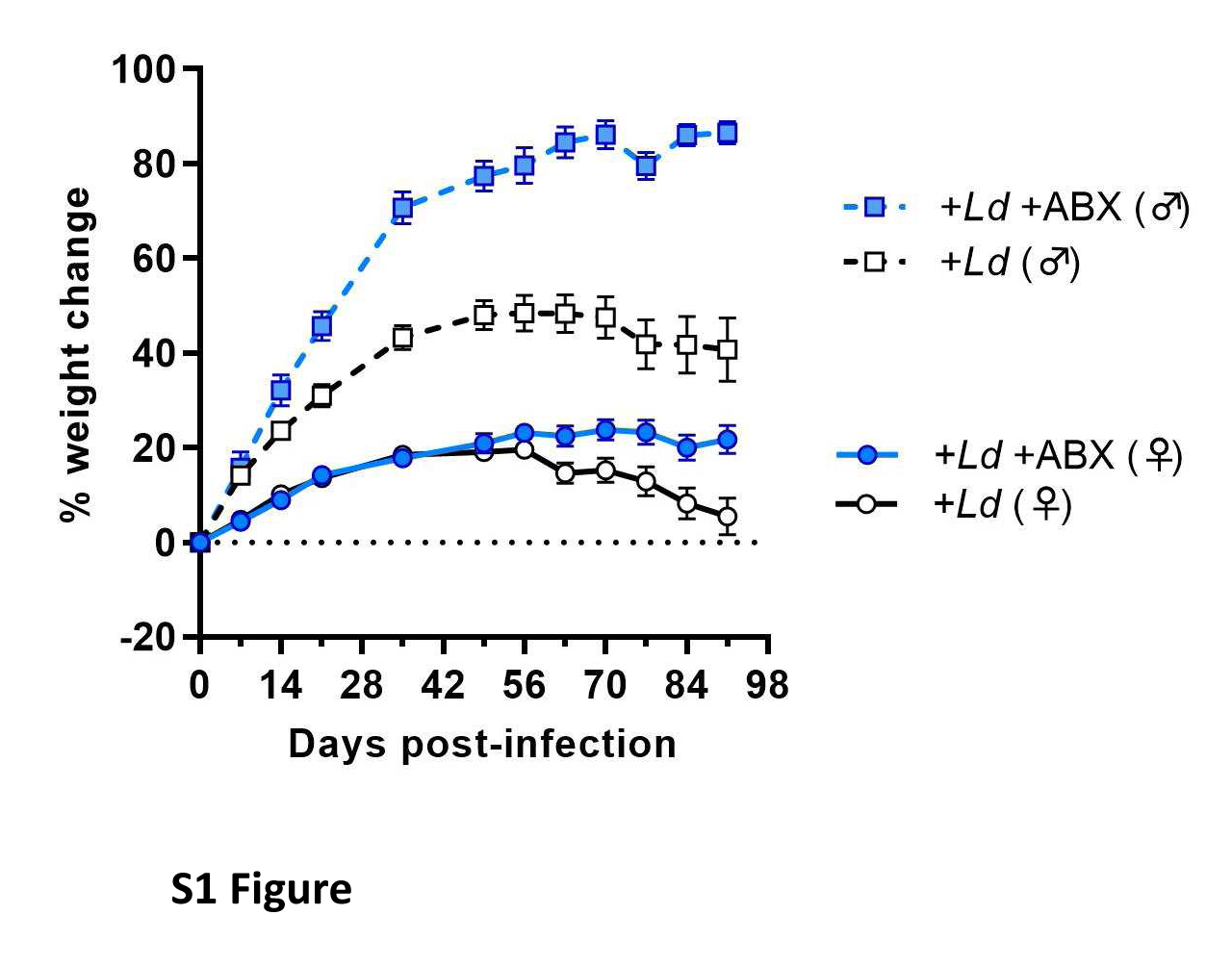

Supplement: S1 Fig — (TIF) [file ppat.1008456.s003.tif]

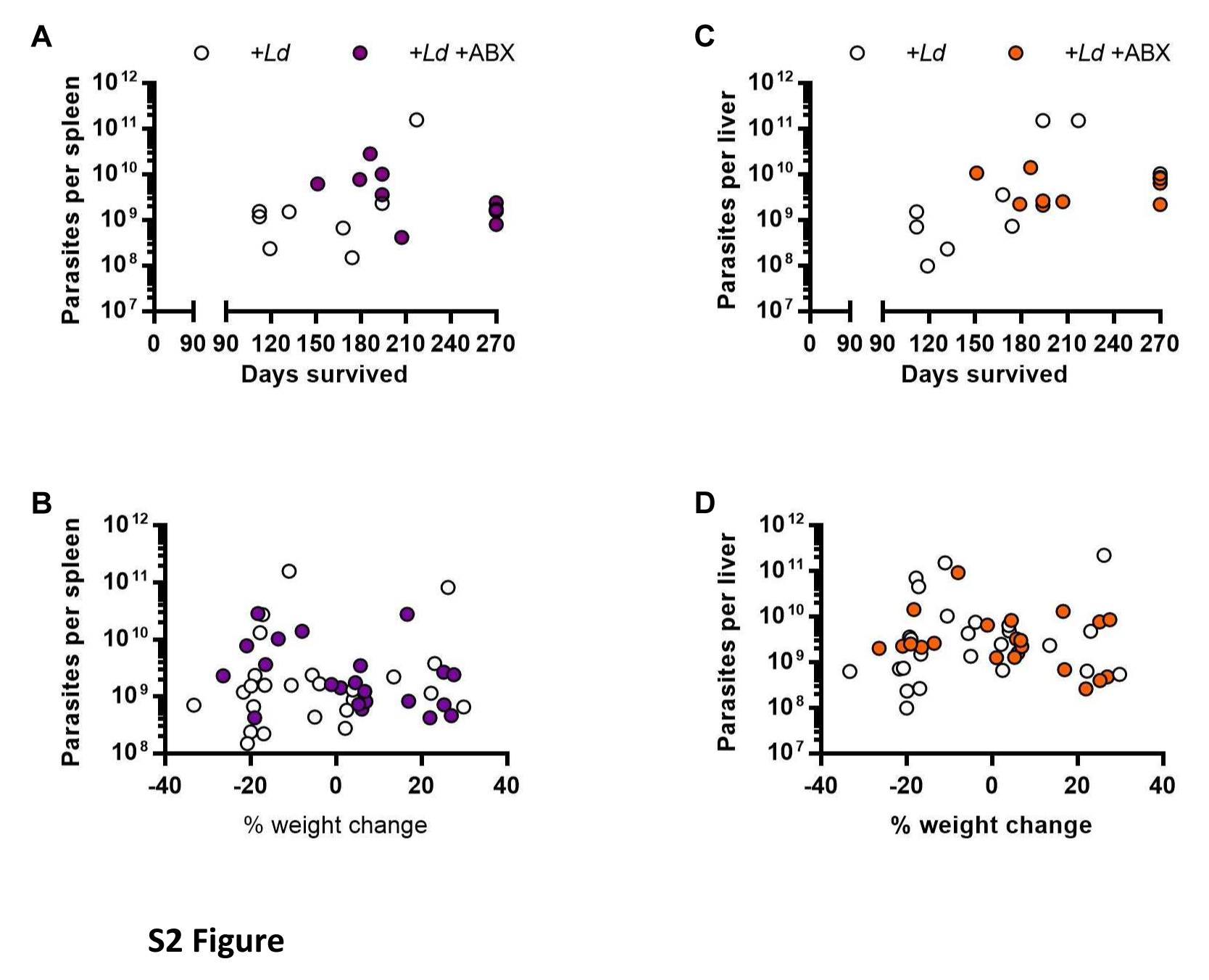

Supplement: S2 Fig — (TIF) [file ppat.1008456.s004.tif]

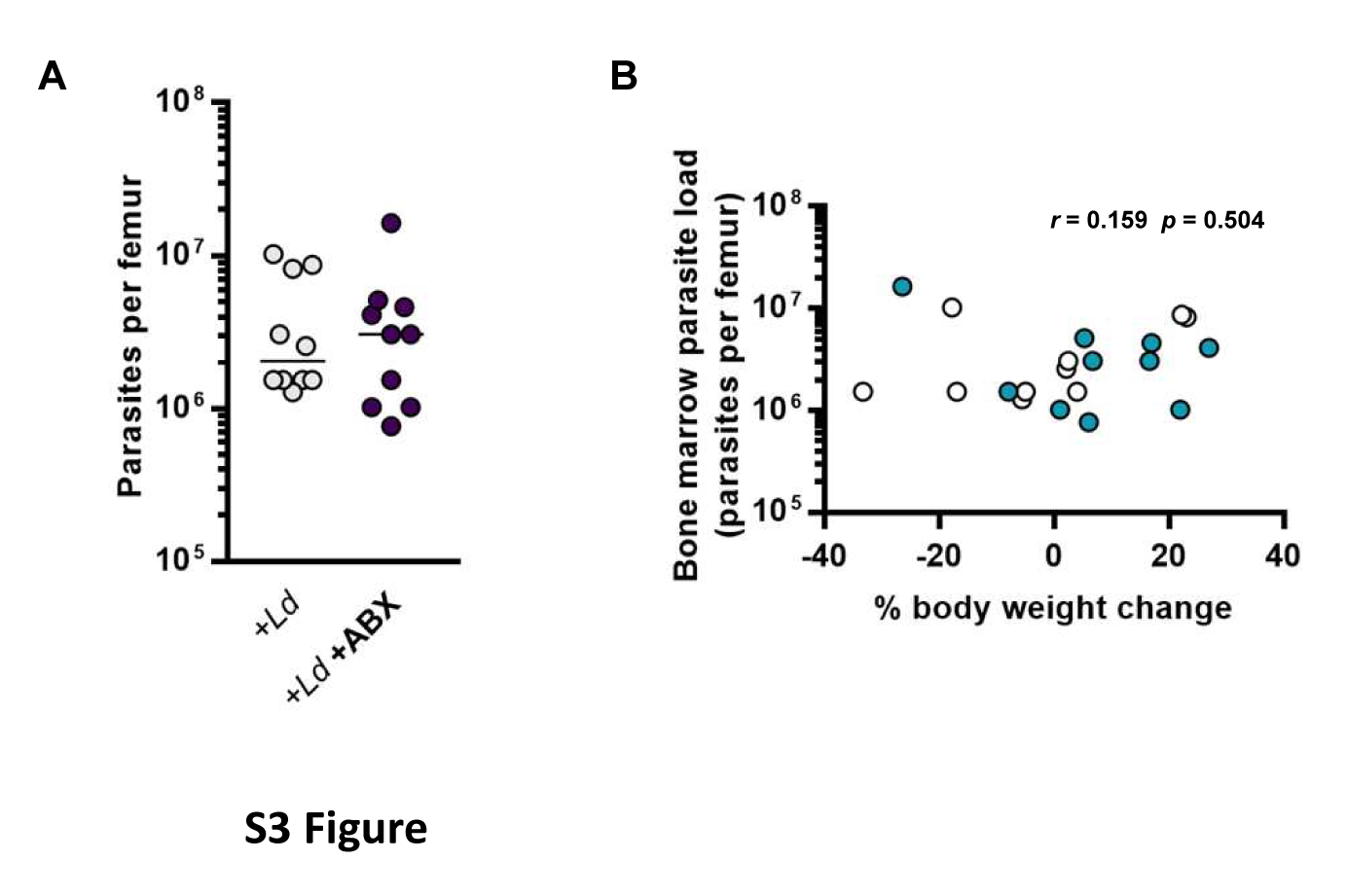

Supplement: S3 Fig — Parasite loads were quantified by limiting dilution in vitro culture assay. (TIF) [file ppat.1008456.s005.tif]

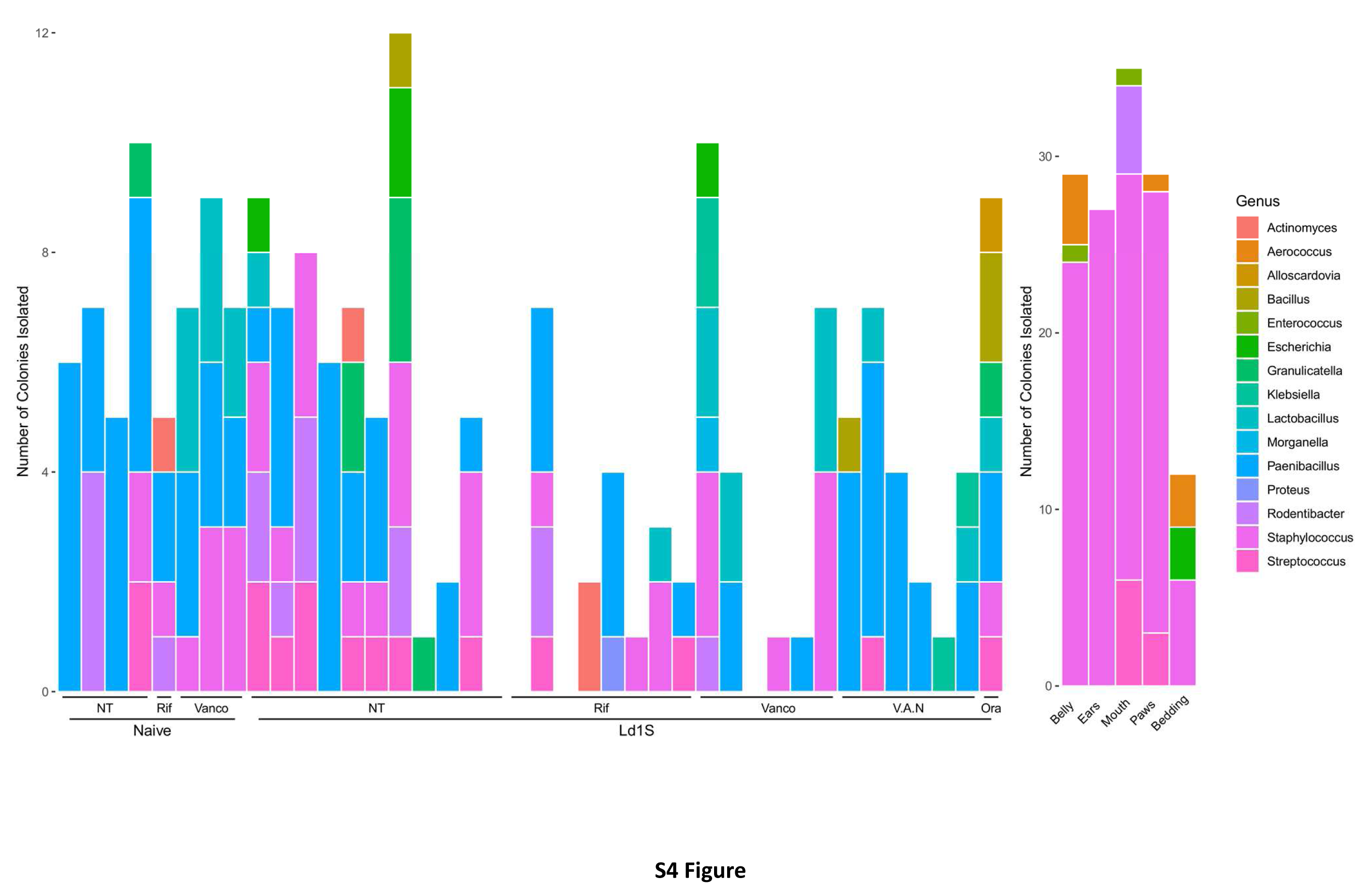

Supplement: S4 Fig — (TIF) [file ppat.1008456.s006.tif]

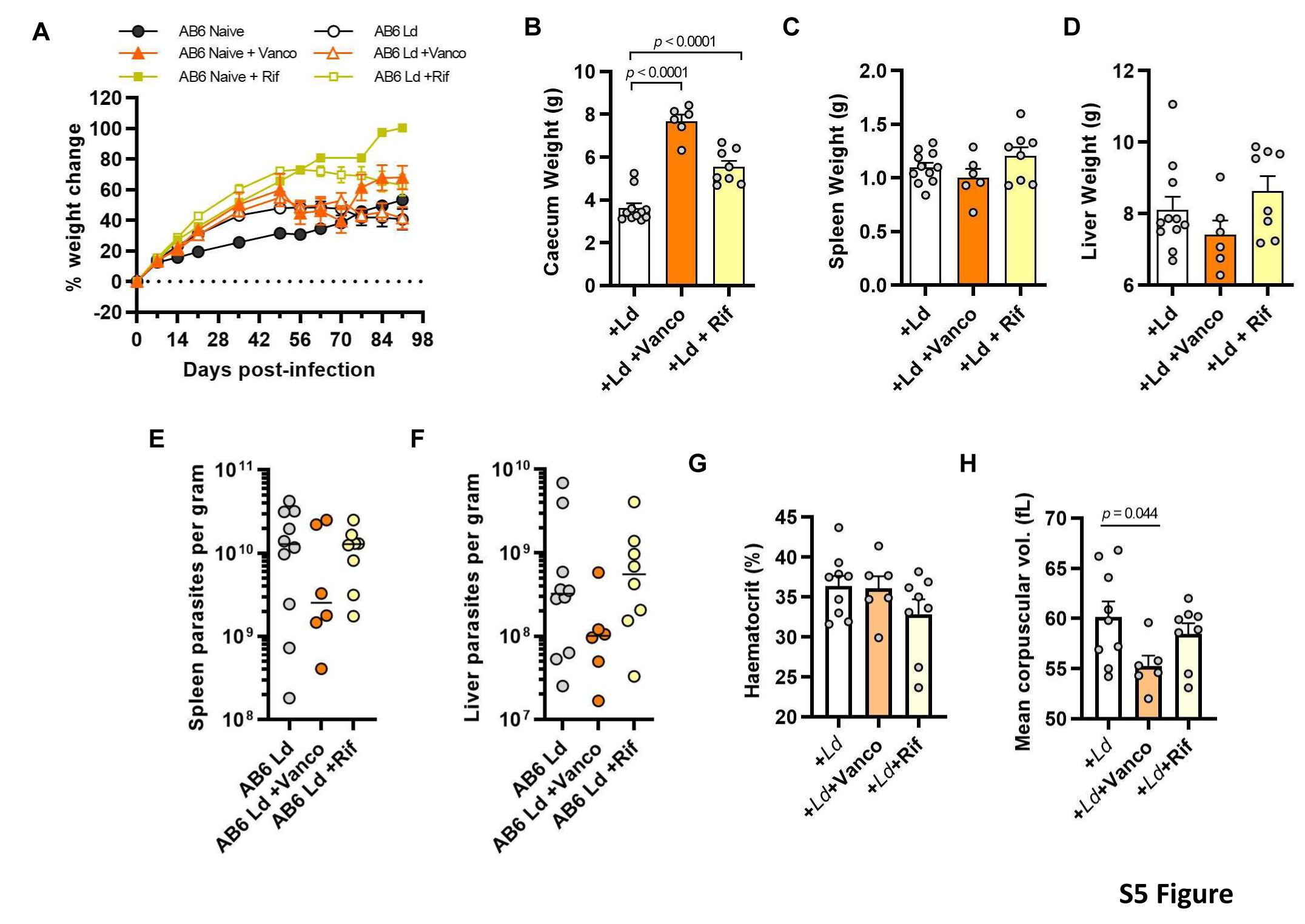

Supplement: S5 Fig — (TIF) [file ppat.1008456.s007.tif]
